# Global seroprevalence of scrub typhus: a systematic review and meta-analysis

**DOI:** 10.1038/s41598-024-61555-9

**Published:** 2024-05-13

**Authors:** Sauvik Dasgupta, Purushothaman Rajamani Asish, Gladys Rachel, Bhavani Shankara Bagepally, Girish Kumar Chethrapilly Purushothaman

**Affiliations:** ICMR-National Institute of Epidemiology, Ayapakkam, Chennai, 600077 India

**Keywords:** Scrub typhus, Global, Prevalence, Systematic review, Meta-analysis, Microbiology, Medical research, Diseases, Infectious diseases

## Abstract

Scrub typhus, a neglected disease, is a significant health concern in the Tsutsugamushi triangle of the Asia–Pacific and has raised global concerns due to recent cases occurring outside this region. To estimate the global prevalence of scrub typhus, we conducted a systematic review and meta-analysis. We conducted a systematic search of PubMed, Scopus, and Embase databases for observational studies on scrub typhus. Using a random-effects model, we combined the prevalence estimates with inverse-variance weights while also evaluating heterogeneity and publication bias. Among 3551 reports screened, we identified 181 studies with 1,48,251 samples for inclusion in our synthesis. The overall pooled seroprevalence (95% confidence intervals) of scrub typhus infections was 24.93% (23.27–26.60). Gender-wise pooled prevalence was estimated to be 50.23% (47.05–53.40) for males and 48.84% (45.87–51.80) for females. Eschar prevalence was observed to be 30.34% (22.54–38.15) among the positive cases. One-fourth of all the samples tested positive for scrub typhus and eschar was present in one-third of these total positive cases, encompassing regions beyond the Tsutsugamushi triangle. This estimation underlines the importance of this neglected disease as a public health problem. Strengthening surveillance and implementing disease control measures are needed in the affected regions.

## Introduction

Scrub typhus, commonly known as Tsutsugamushi disease, is an acute febrile illness caused by an obligatory intracellular gram-negative α-proteobacterium *Orientia tsutsugamushi*^1–3^*.* Since the initial descriptions dated as early as 313 AD in China^4^, scrub typhus continues to be a major zoonosis^5^. The disease is spread through the bite of trombiculid mites infected with *O. tsutsugamushi*^1^*.* After 5–14 days following exposure to the bite of an infected vector, the person develops a flu-like illness with symptoms such as fever, rash, eschar at the bite site, headache, myalgia, cough, widespread lymphadenopathy, nausea, vomiting, rash, and abdominal discomfort^1,6^. A high fatality rate is seen among scrub typhus cases hospitalized with pulmonary, cardiac, hepatic, neurological, or renal complications^7,8^.

Scrub typhus is endemic to the area in the Asia–Pacific and Northern Australia region, referred to as the 'Tsutsugamushi Triangle'. To date, more than 70 strains of *O. tsutsugamushi* have been reported including the prototypical strains of Karp, Gilliam, and Kato^1,6^. The clinical features observed in infected individuals are strain-specific and depend on their levels of virulence^9–11^.

Globally, about a million cases of scrub typhus are reported each year, with a case fatality rate of 30% or higher if left untreated^12^ with variations observed in countries. The case fatality rates vary across different regions, with China recording a rate of 13.8%^13^, while Japan shows a notably lower rate of 1%^14^. South Korea reports a rate of 6.3%^15^, Thailand at 13.6% [Supplementary ref S1: 77], and India with a rate of 33.3% [Supplementary ref S1: 122]. Understanding the global prevalence of scrub typhus is essential for effective disease control and prevention strategies^16^. Scrub typhus prevalence studies are distributed over different geographical regions, with varying methodologies and sample sizes. This distribution makes it difficult to assess the overall burden of the disease. A systematic review and meta-analysis would provide synthesized evidence of available data, allowing for a global estimation of scrub typhus prevalence.

## Methods

### Search and selection of studies

We included observational studies (cross-sectional and cohort) published in English to estimate the global prevalence of scrub typhus infections. The study protocol was registered on PROSPERO database (Reg. No. CRD42021234124) and reported according to the Preferred Reporting Items for Systematic Reviews and Meta-Analysis (PRISMA) guidelines^17^. We searched PubMed, Scopus, and Embase, databases from the inception of each database up to March 11, 2024. The search strategies for each database is provided in the Supplementary Table S1. Articles involving in vitro studies, abstracts, case reports, reviews, theses, books, discussions, opinion pieces, modeling studies, conference abstracts, and editorials were excluded.

Two reviewers conducted the screening. After the search, all downloaded literature was imported into Rayyan software, and duplicates were eliminated^18^. Rayyan is a cloud-based software application designed for systematic reviews and other types of literature reviews (Manufacturer: Rayyan Systems Inc., Doha, Qatar). We further performed the title and abstract screening. The abstracts of studies that reported scrub typhus cases were considered eligible for full-text review. Two reviewers selected studies from eligible studies using the eligibility criteria. Discrepancies were discussed and resolved by consensus after consulting the third reviewer.

### Study participants

The participants of included studies were individuals positive for scrub typhus infection, based on Immunoglobulin M or G (IgM/ IgG) detection using Enzyme-linked immunosorbent assay (ELISA), immunofluorescence assay (IFA), indirect immune peroxidase assay (IIPA), rapid diagnostic test (RDT), Weil-Felix test, or Polymerase chain reaction (PCR).

### Data extraction and management

The two reviewers independently extracted information from the selected studies using a pre-designed data extraction form in Microsoft Excel. Extracted data included general information (author, study title, publication year, country), method information (study design, study setting, type of population, gender, study location, study period, laboratory investigations), and result information (number of Scrub typhus positives). Disagreements among the reviewers were resolved through discussion and consensus after consulting the third reviewer which has been elaborated in the Supplementary material S1 (page 50)^19^. The extracted data were analyzed using STATA software version 17^20^.

### Risk of bias assessment

We used the AXIS tool^21^ and Newcastle–Ottawa scale (NOS)^22^ to assess the quality of analytical cross-sectional and cohort studies, respectively. The AXIS tool is designed to measure the aspects of study quality with 20 items, including justification of sample size, representativeness of the sample, a description of non-responders, use of validated measures, description of statistical methods, discussion of non-response bias and reporting of funding and conflicts of interest. The AXIS tool assesses the individual characteristics of a study cumulatively but not with a quantitative score. The risk of bias judgment was made through signaling questions and responses, namely "yes", "no", and "don't know". Quality evaluation through NOS was done by assigning up to nine items for the least risk of bias in three domains: (1) selection of study group (four items), (2) comparability of groups (two items) and (3) ascertainment of exposure and outcomes (three items) for cohort studies, respectively. Two authors independently assessed the risk of bias for each included study, and disagreements were discussed and resolved by consensus.

### Statistical analysis

The pooled prevalence using the random effects (DerSimonian and Laird) method was reported as a proportion with a 95% confidence interval (CI). I^2^ test was used to assess the evidence of heterogeneity. I^2^ value < 25% was considered mild, 25–75% moderate, and > 75% was regarded as having substantial heterogeneity^23^. We used a Forest plot to represent the study-specific and pooled estimates for overall and subgroup analysis. A funnel plot was constructed to examine the publication bias (Supplementary Fig. S1). The subgroup analysis was conducted, and the pooled prevalence of Scrub typhus infections was estimated according to the study populations, study types, study settings (hospital vs. community; seasonal vs. non-seasonal), study areas, recent infections and past exposures.

### Ethical approval

The study does not require ethical approval as it involves meta-analysis of publicly available research and utilized anonymized original data.

## Results

**Figure 1:**
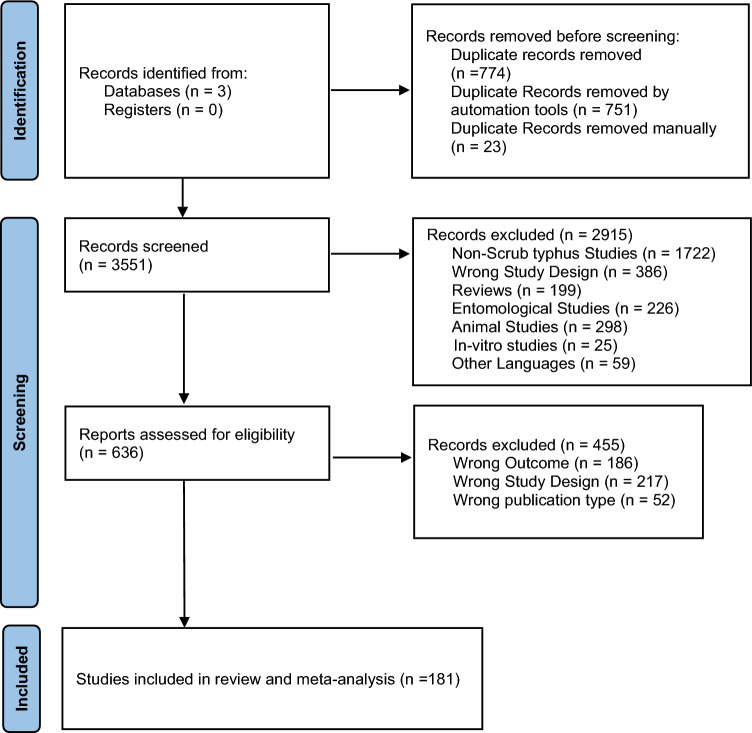
PRISMA flow chart of study selection.

After reviewing full-text studies, 181 studies [Supplementary reference S1: 1–181] published between 1972 and 2024, including twelve multicentric studies [Supplementary reference S1: 23, 27, 47, 50–52, 62, 63, 68, 77, 141, 172], were finally included in this systematic review for narrative synthesis analysis (Fig. 1).

### Characteristics of the studies

The characteristics of the included 181 studies are summarized in Supplementary Table S2. The studies were published between 1972 and 2024, mostly from Asia (n = 168) [Supplementary reference S1: 4, 5, 7, 9, 11, 21, 24, 26, 28–33, 35–39, 41, 42, 44–56, 58–64, 66–68, 70–124, 126–132, 134, 135, 138–141, 142–161, 163–165, 167–181], followed by Africa (n = 4) [Supplementary reference S1: 43, 65, 125, 141], Australia (n = 3) [Supplementary reference S1: 27, 34, 40], South America (n = 4) [Supplementary reference S1: 57, 137, 162, 166], Europe (n = 1) [Supplementary reference S1: 24] and North America (n = 1) [Supplementary reference S1: 19]. The 181 studies included 174 prospective and thirteen retrospective studies covering 197 study groups. Among the studies, 147 were hospital-based, 31 were community-based, and three studies involved hospital and community components. Urban and rural (mixed) populations were covered in 152 studies, whereas twenty-three studies specifically focused on rural populations and the remaining six studies involved urban populations exclusively. In terms of the clinical spectrum, 68.75% of all patients exhibited Acute Febrile (AFI)/Acute Undifferentiated Febrile Illness (AUFI), 2.42% experienced Acute Encephalitis Syndrome (AES), 0.65% suffered from Acute Kidney Injury (AKI) and 0.43% of the total patients exhibited Acute Respiratory Distress Syndrome (ARDS) respectively. Fatalities among cases were observed in thirty (16.57%) studies.

Based on the type of study, eleven were cohort studies [Supplementary reference S1: 4, 39, 45, 48, 70, 77, 152, 171, 172, 178, 179] comprising seven prospective and four retrospective cohorts. Seven of the cohort studies looked into patients with current infection [Supplementary reference S1: 4, 39, 48, 70, 152, 178, 179] and three looked into patients with previous exposure to scrub typhus respectively [Supplementary reference S1: 45, 77, 171, 172]. The 170 cross-sectional studies [Supplementary reference S1: 2–4, 6–19, 21–24, 26–42, 44–63, 65–89, 91–119, 121–129, 132–151, 153–170, 173–177, 180–181] comprised 185 study groups. Thirty-one studies were community-based [Supplementary reference S1: 3, 5, 6, 9, 10, 13, 18, 19, 22, 23, 25, 27, 28, 40, 42, 53, 69, 77, 79, 111, 120, 128, 136, 155–157, 162, 164, 166, 172, 174]. Exclusively hospital-based studies were 147 [Supplementary reference S1: 1, 2, 4, 7, 8, 11, 12, 14–17, 20, 21, 24, 26, 29–31, 33–36, 38, 39, 41, 43–52, 54–68, 70–78, 80–110, 112–119, 121–127, 129–135, 137–142, 144–154, 158–161, 163, 165, 167–171, 173, 175–181]. Three studies had both hospital and community settings [Supplementary reference S1: 21, 137, 143].

Based on study population type, 144 studies involved patients [Supplementary reference S1: 1, 2, 4, 7, 8, 11, 12, 14–17, 19–21, 24, 26, 28–36, 38–41, 43, 44, 46–52, 54–68, 71–76, 78, 81–85, 87–100, 102–110, 112–119, 121–127, 129–135, 138–140, 142–147, 149–154, 158–161, 163, 165–171, 173, 175–180, 181], twenty-three studies were on healthy individuals [Supplementary reference S1: 3, 5, 6, 9, 10, 13, 18, 22, 27, 37, 42, 53, 69, 77, 79, 86, 120, 128, 155, 156, 157, 162, 174], four studies involved pregnant women [Supplementary reference S1: 69, 80, 101, 137], three studies included military personnel [Supplementary reference S1: 20, 50], three studies involved tribal populations [Supplementary reference S1: 23, 136, 164], two studies involved pediatric population [Supplementary reference S1: 70, 179], one study involved refugees [Supplementary reference S1: 25] and one study each covered blood donors [Supplementary reference S1: 17], and travelers [Supplementary reference S1: 24].

Based on the signs and symptoms of the patients involved, 151 studies with 164 study groups reported fever as the inclusion/enrolment criteria [Supplementary reference S1: 2–4, 6–16, 19, 21–26, 29–32, 34, 35, 37–40, 42, 44–47, 50–53, 56, 57, 59–75, 77, 78, 80, 82, 84, 85, 87–95, 98, 100, 101, 103–107, 110–113, 116, 121–126, 128, 129, 131, 132, 134–136, 138–140, 141–154, 158–161, 163, 165–172, 173, 176–181], fifty-six studies included co-infections such as spotted fever, malaria, dengue, murine typhus, kala-azar, etc. [Supplementary reference S1: 2, 8, 17, 18, 20, 21, 29, 30, 33, 35, 36, 39, 40, 42, 45, 47, 51, 52, 54, 57, 58, 60, 62, 66, 74–76, 79, 80, 82–84, 86, 89, 94, 96, 98, 101, 112, 114, 115, 121, 123, 125, 143, 147, 149, 157, 159, 161, 165, 174, 176–178]. Twenty-seven groups from twenty-five studies confirmed the presence of *O. tsutsugamushi* strains [Supplementary reference S1: 8, 15, 33, 44, 55, 62, 76, 79, 100, 104, 105, 107, 108, 110, 113, 141, 142, 148, 154, 158, 161–163, 168, 175].

The distribution of study participants' age was available in only 173 studies. Fourty-nine studies (27.07%) reported the mean participant age to be above fourteen years [Supplementary reference S1: 2, 6, 9, 18, 24, 26, 27, 30, 37, 39, 40, 45, 47, 49, 54, 55, 56, 59, 69, 70, 80, 84, 88, 90, 93, 99, 102, 110, 111, 112, 115, 119, 121, 123, 127, 129, 136, 139, 140, 142, 144, 146, 147, 150, 154, 157, 169, 172, 177]. In studies among children, the age ranged from birth (neonates) to twelve years. Age-related information was missing in eight studies.

Sero-positivity rates for different age groups were calculated and tabulated in Supplementary Table S3. Scrub typhus with coinfections was reported in fifty-six studies (Supplementary Table S4). The studies used a wide range of serological (non-specific and specific) and molecular techniques viz, Weil-Felix/ELISA/RDT/PCR/IFA/IIPA for scrub typhus detection (Supplementary Table S5). Twenty-five studies reported the type of *O. tsutsugamushi* strains. (Supplementary Table S6).

### Risk of bias assessment

The quality of the studies assessed in this review ranged from low to moderate, with all 170 quantitative studies not meeting 8 out of 20 possible criteria of the AXIS tool. Of the studies that missed eight items, most did not take measures to address the concerns of non-response bias and outcome variables measured in accordance with the aims of the study. One hundred and fifty-nine studies (93.5%) lacked sample size estimation. One hundred and sixty-nine studies (99.4%) used a sample frame from a population closely representing the target or reference population. All the studies satisfied 12 of the 20 major criteria as per the AXIS tool (Supplementary Table S7). Newcastle–Ottawa quality assessment of cohorts rated most of the included studies to be good (Supplementary Table S8).

### Meta-analysis

**Figure 2:**
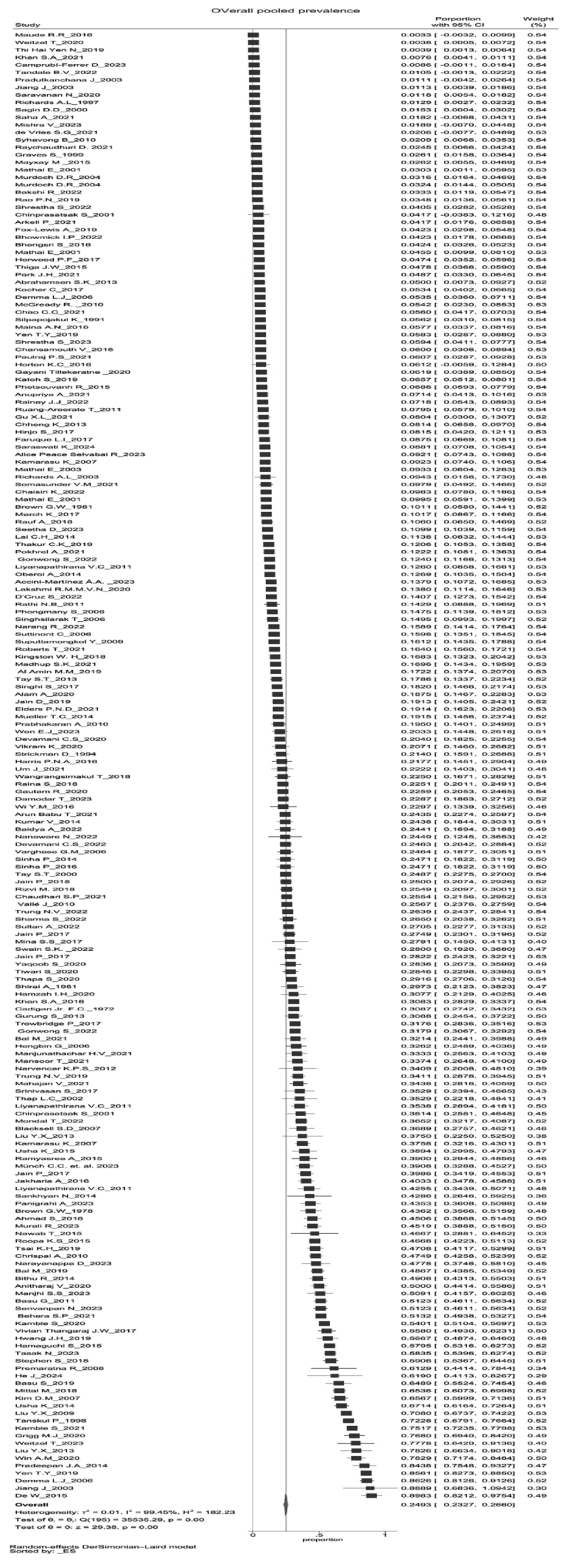
Overall pooled prevalence of Scrub typhus.

Based on the 181 studies, the pooled prevalence of scrub typhus was estimated to be 24.93% (23.27–26.60) (Fig. 2) which is around one-fourth of the population included in our meta-analysis. We found substantial heterogeneity between the studies that reported the outcome (I^2^ = 99.45%). Based on gender, the pooled prevalence of scrub typhus among males was 50.23% (47.05–53.40), whereas, among females, it was 48.84% (45.87–51.80) (Supplementary Figs. S2, S3).

Scrub typhus is typically characterized by an eschar, considered the most useful diagnostic evidence for the disease^16^. Eschar positivity in the pooled studies was estimated to be 30.34% (22.54–38.15) however, the estimates varied between countries. The highest prevalence of eschar was recorded from South Korea [89.21% (83.68–94.75)] and the lowest was recorded from Australia [3.70% (3.42–10.83)] (Supplementary Fig. S4). The pooled prevalence of scrub typhus infections during the seasonal periods was 43.95% (27.83–60.06), whereas it was 23.43% (21.82–25.04) during non-seasonal periods (Supplementary Figs. S5, S6).

### Subgroup analysis

The estimated pooled prevalence of scrub typhus infections among various study populations are as follows: pediatric 45.19% (38.88–51.50), pregnant women 31.70% (12.59–75.98), tribals 25.87% (7.83–59.58), patients 25.60% (23.65–27.54), healthy population 22.29% (18.19–26.39), soldiers 22.60% (10.99–34.21), refugees 22.22% (14.03–30.41), blood donors 10.11% (5.80–14.41), and travelers 0.99% (0.07–1.91) (Supplementary Fig. S7). We also estimated the prevalence of scrub typhus in community-based [27.60% (22.89–32.31)] and hospital-based studies [23.80% (22.16–25.43)] (Supplementary Figs. S8, S9). The pooled prevalence of scrub typhus based on the study areas was 30.44% (20.33–40.55) in urban areas, 27.73% (20.68–34.77) in rural areas, and 24.37% (22.65–26.08) in studies involving both urban and rural areas (Supplementary Fig. S10). The pooled prevalence estimates of scrub typhus infections among different countries are shown in Supplementary Fig. S11. The estimated pevalence of AUFI/AFI among patients affected with scrub typhus was 24.95% (23.06–26.84), AES was 33.31% (23.75–42.87), AKI was 22.25% (7.93–36.58) and ARDS was 12.12% (0.35–28.89) respectively (Supplementary Fig. S12).

Studies were grouped based on the exposure status of the study population, i.e., currently infected (IgM) and previously exposed (IgG) populations.

### Scrub typhus among currently infected (IgM positive) populations

Pooled data from twenty-two studies with current infections based on IgM showed the seroprevalence to be 51.97% (32.47–71.46) (Supplementary Fig. S13). Based on the study settings, such as community-based and hospital-based, the pooled prevalence was estimated to be 35.94% (22.15–49.73) and 62.21% (45.31–79.12) respectively (Supplementary Figs. S14, S15). Similarly, based on the type of population, the IgM prevalence was 55.37% (31.74–79.00) in patients, 32.79% (28.62–36.95) among pregnant women, 32.51% (27.05–37.97) among tribal population and 20.82% (7.33–48.97) in healthy-population (Supplementary Fig. S16). The country-wise seroprevalence of Scrub typhus among the currently infected cases was: Iraq [92.86% (83.32–102.40), Nepal [61.75% (49.81–73.69)], India [56.38% (28.33–84.43)], Indonesia [51.65% (41.38–61.92)], Thailand [33.89% (30.02–37.75)], Democratic Republic of Sao Tome and Principe [32.79% (28.62–36.95)], and China [18.17% (14.90–21.44)] (Supplementary Fig. S17). The estimated pooled prevalence of current infection among both rural and urban areas was 57.14% (37.90–76.39), whereas in exclusively rural settings was 33.75% (18.17–49.33) (Supplementary Fig. S18). There were no studies conducted exclusively in urban areas.

### Scrub typhus infection among previously exposed (only IgG positive) populations

Among the previously infected (IgG) group, the pooled seroprevalence was 57.86% (40.34–75.38) from thirteen studies (Supplementary Fig. S19). The pooled prevalence in community and hospital settings was estimated to be 74.96% (58.08–91.84) and 40.63% (6.51–74.74) respectively (Supplementary Figs. S20, S21). Based on the type of population, the IgG prevalence in healthy populations was 77.39% (63.66–91.13); among patients, 52.58% (29.68–75.48) and 27.46% (23.50–31.42) among pregnant women (Supplementary Fig. S22). The country-wise seroprevalence of past exposure to Scrub typhus was: Colombia [92.54% (86.24–98.83)], India [83.20% (76.60–89.80)], Thailand [65.41% (59.96–70.87)], Palau [64.33% (56.84–71.82)], Indonesia [49.45% (39.18–59.72)], Democratic Republic of Sao Tome and Principe [27.46% (23.50–31.42)], China [13.84% (5.74–33.41)], and Vietnam [9.71% (3.99–15.43)] (Supplementary Fig. S23). We also estimated the seroprevalence of IgG among the different study areas. The estimated pooled prevalence among both rural and urban areas was 46.32% (21.88–70.76), whereas, in exclusively rural settings, it was 84.94% (74.88–95.00), and 92.54% (86.24–75.38) in urban areas respectively (Supplementary Fig. S24).

The presence of both IgM and IgG antibodies were estimated to be 15.38% (6.58–24.19) among the positive patients.

## Discussion

This systematic review and meta-analysis aims to provide a comprehensive quantitative assessment of the global prevalence of Scrub typhus infections. This meta-analysis incorporates diverse populations, such as blood donors, healthy volunteers, households, children, travelers, and pregnant women, affected by scrub typhus. The results of our analysis reveal a pooled prevalence rate of 24.93%, indicating that roughly one-fourth of all febrile cases worldwide are positive for scrub typhus. Moreover, our examination of previously exposed populations yielded an overall seroprevalence of 57.86%, suggesting that approximately half of the individuals tested had prior exposure to *O. tsutsugamushi*. The majority of cohort studies analyzed in our study reported positivity using IgM, indicating prevalence. However, four cohort studies reported scrub typhus based on IgG, potentially leading to an underestimation of prevalence. Furthermore, given that only three cohort studies reported scrub typhus based on IgG, the impact on the pooled prevalence is likely to be minimal. A variability in country-wise seroprevalence among the currently infected population was evident, with Iraq exhibiting notably high seroprevalence. This finding was based upon a rural community which had consistent interaction with animal reservoirs. Intriguingly, our analysis did not discern any significant gender predilection, as infection rates stood comparable between males and females. Furthermore, our estimations indicate that approximately 30% of individuals diagnosed with scrub typhus exhibit the presence of eschars, with variations observed across different countries highlighted in the Supplementary Fig. S4. The variations seen within countries and regions are directly related to how methodical and diligent the physicians were in examining their patients. Additionally, our analysis estimated a quarter of the tested population across different settings, including hospitals and communities, as well as in various study areas, encompassing urban, rural, and urban–rural regions to be exposed to scrub typhus infection.

The majority of studies in our analysis come from endemic (Tsutsugamushi triangle) and regions which earlier had not reported scrub typhus. Globalization and increased travel have expanded the reach of scrub typhus into areas earlier not known for reporting scrub typhus^16,24^, often presenting with nonspecific clinical symptoms. Limited awareness and diagnostic resources likely contribute to significant underreporting of scrub typhus cases, potentially explaining lower seroprevalence in island nations compared to others. Eschar presence varies widely (7% to 97%) among scrub typhus patients and the absence of eschar is reported to be an independent predictive factor for fatal outcomes^25^. Variability in eschar occurrence is noted across different *O. tsutsugamushi* strains^10,26^ and among different ethnic populations^27,28^. Community-based studies are crucial for assessing disease exposure risk. Workplace and socioeconomic status are significant risk factors for human scrub typhus. Agricultural activities pose the primary risk for both rural and urban populations [Supplementary reference S1: 58]. Deforestation and development projects lead to secondary growth of scrub vegetation, increasing mite and rodent populations [Supplementary reference S1: 28, 52, 112]. Risk factors include housing type, rodents in peri-domestic areas, and forest interactions, with higher prevalence in rural areas [Supplementary reference S1: 107]. Scrub typhus which was once thought to be a rural illness, is being increasingly recognized and diagnosed in urban settings [Supplementary reference S1: 75,108]. The large population could have a significant effect on urban scrub typhus. Large urban populations, with their dense human presence, poor sanitation, and encroachment into natural habitats, coupled with environmental changes and increased man vector contact, create ideal conditions for the spread of scrub typhus. Urban scrub typhus should be regarded as one of the differential targets for febrile illness in areas where it is endemic. Urban environments differ from their rural surroundings. The eco-friendly trend of creating more green spaces such as parks in urban areas provides a conducive environment for rodent infestation resulting in such spaces acting as potential sites for scrub typhus transmission^29^.

Our review had some limitations that should be considered when interpreting the results. The high degree of heterogeneity observed among the included studies could have impacted the generated estimates. This heterogeneity could possibly be attributed to various factors, such as variations in the ethnicity of the study populations and differences in study methodologies, particularly design, sampling and diagnostic methods. Utilization of four cohort studies to report scrub typhus based on IgG may have contributed to an underestimation of the pooled prevalence. Few studies were included under different sub-groups, which might have overestimated their seroprevalence. Studies reporting equal numerator and denominator values were also excluded from the meta-analysis due to considerations of selection bias. Grey literature comprising academic papers, reports, dissertations, ongoing yet unpublished research as well as preprints were not included in our review. The exclusion of non-English publications might have resulted in a language selection bias in the study.

## Conclusion

This systematic review and meta-analysis of the prevalence of scrub typhus highlights the worldwide spread of the disease. One-fourth of the population included in our meta-analysis were found to be affected by scrub typhus, with eschar being detected in about one-third of the scrub typhus confirmed cases. Males and females are at equal risk of getting infected. These findings extend to regions beyond the Tsutsugamushi triangle. This expansion beyond the endemic areas emphasizes the need for enhanced surveillance involving those regions. Monitoring disease trends and deploying public health interventions targeting vector and rodent control in the endemic areas is needed to tackle *O. tsutsugamushi* transmission to reduce incident cases.

### Supplementary Information

Supplementary Information.

## Data Availability

The data extracted from the included studies and data used for analysis are provided in the supplementary material.
