# Supplementary material for: Global seroprevalence of scrub typhus: a systematic review and meta-analysis
Source: Sci Rep. 2024 May 13;14:10895. doi: 10.1038/s41598-024-61555-9 (PMC11091130; doi:10.1038/s41598-024-61555-9)
Supplement: Supplementary file 1 — Supplementary Information. [file 41598_2024_61555_MOESM1_ESM.docx]

| **Sl no** | **Content** | **Page no** |
| --- | --- | --- |
|  | Supplementary References | 3-15 |
|  | Supplementary Table 1. Search words | 16 |
|  | Supplementary Table 2. Characteristics of the studies. | 17-20 |
|  | Supplementary Table 3: Proportion positivity of scrub typhus among different age-groups | 21 |
|  | Supplementary Table 4: Studies reporting scrub typhus with co-infections | 21 |
|  | Supplementary Table 5: Diagnostic tests used for detecting scrub typhus among different studies | 22 |
|  | Supplementary Table 6: Strains reported from scrub typhus positive patients | 22 |
|  | Supplementary Table 7. Risk of bias assessment for Cross-sectional studies. AXIS TOOL | 23-26 |
|  | Supplementary Table 8. Risk of bias assessment for Cohort studies. NEW-CASTLE OTTAWA SCALE | 27 |
|  | Supplementary Figure 1. Funnel plot | 28 |
|  | Supplementary Figure 2. Pooled prevalence of Scrub typhus among males | 29 |
|  | Supplementary Figure 3. Pooled prevalence of Scrub typhus among females | 30 |
|  | Supplementary Figure 4. Pooled prevalence of eschar among scrub typhus cases | 31 |
|  | Supplementary Figure 5. Pooled prevalence of Scrub typhus during seasonal | 32 |
|  | Supplementary Figure 6. Pooled prevalence of Scrub typhus during non-seasonal | 33 |
|  | Supplementary Figure 7. Pooled prevalence of Scrub-typhus among sub-group (Population-wise) | 34 |
|  | Supplementary Figure 8. Pooled prevalence of Scrub-typhus among sub-group (Study-setting: Community) | 35 |
|  | Supplementary Figure 9. Pooled prevalence of Scrub-typhus among sub-group (Study-setting: Hospital) | 36 |
|  | Supplementary Figure 10. Pooled prevalence of Scrub-typhus among sub-group (Study-localities) | 37 |
|  | Supplementary Figure 11. Pooled prevalence of Scrub-typhus among sub-group (Country-wise) | 38 |
|  | Supplementary Figure 12. Pooled seroprevalence of symptoms among Scrub typhus cases | 39 |
|  | Supplementary Figure 13. Pooled prevalence of Scrub-typhus among currently infected (based on IgM) | 40 |
|  | Supplementary Figure 14. Pooled prevalence of Scrub-typhus among currently infected in community-settings | 41 |
|  | Supplementary Figure 15. Pooled prevalence of Scrub-typhus among currently infected in hospital-settings | 41 |
|  | Supplementary Figure 16. Pooled prevalence of Scrub-typhus among currently infected in different populations | 42 |
|  | Supplementary Figure 17. Country-wise pooled prevalence of Scrub-typhus among currently infected | 43 |
|  | Supplementary Figure 18. Pooled prevalence of Scrub-typhus among currently infected from different study localities | 44 |
|  | Supplementary Figure 19. Pooled prevalence of Scrub-typhus among previously exposed | 45 |
|  | Supplementary Figure 20. Pooled prevalence of Scrub-typhus among previously exposed in community settings | 45 |
|  | Supplementary Figure 21. Pooled prevalence of Scrub-typhus among previously exposed in hospital settings | 46 |
|  | Supplementary Figure 22. Pooled prevalence of Scrub-typhus among previously exposed in different populations | 47 |
|  | Supplementary Figure 23. Country-wise pooled prevalence of Scrub-typhus among previously exposed | 48 |
|  | Supplementary Figure 24. Pooled prevalence of Scrub-typhus among previously exposed from different study localities | 49 |
|  | Note on managing reviewer disagreements | 50 |

Supplementary References

1. Abrahamsen SK, Haugen CN, Rupali P, Mathai D, Langeland N, Eide GE, et al. Fever in the tropics: Aetiology and case-fatality - a prospective observational study in a tertiary care hospital in South India. BMC Infect Dis. 2013;13:1–8.
2. Ahmad S, Dhar M, Mittal G, Bhat NK, Shirazi N, Kalra V, et al. A comparative hospital-based observational study of mono- and co-infections of malaria, dengue virus and scrub typhus causing acute undifferentiated fever. Eur J Clin Microbiol Infect Dis. 2016;35:705–11.
3. Al Amin MM, Paul SK, Aung MS, Paul A, Aziz MA, Khan NA, et al. Molecular characterization of Orientia tsutsugamushi causing scrub typhus among febrile patients in north-central Bangladesh. New Microbes New Infect. 2019;32:100595–100595.
4. Alam A, Agarwal P, Prabha J, Jain A, Kalyan RK, Kumar C, et al. Prediction Rule for Scrub Typhus Meningoencephalitis in Children: Emerging Disease in North India. J Child Neurol. 2020;35:820–7.
5. Anitharaj V, Stephen S, Pratheesh P. Scrub typhus in Puducherry, India: Application of nested PCR targeting three different genes - 56 kDa, 47 kDa and groEL of Orientia tsutsugamushi and comparison with ST IgM ELISA. J Vector Borne Dis. 2020;57:147–52.
6. Anupriya A, Rajkumar B, Prabhusaran N, Priya BS. Scrub Typhus Infection Among PUO Cases in the Pediatric Population in a Tertiary Care Hospital. Asian J Pharm Clin Res [Internet]. 2021;14:137–9. Available from: <http://dx.doi.org/10.22159/ajpcr.2021v14i5.41143>.
7. Babu TA, Narayanasamy DK, Jamir L. Prospective study to assess the response to therapy and its predictors in children with scrub typhus. J Trop Pediatr. 2021;67.
8. Bal M, Mohanta MP, Sahu S, Dwibedi B, Pati S, Ranjit M. Profile of Pediatric Scrub Typhus in Odisha, India. Indian Pediatr. 2019;56:304–6.
9. Basu G, Chrispal A, Boorugu H, Gopinath KG, Chandy S, Prakash JAJ, et al. Acute kidney injury in tropical acute febrile illness in a tertiary care centre RIFLE criteria validation. Nephrol Dial Transplant. 2011;26:524–31.
10. Basu S, Saha A, Sarkar S, Sinha MK, Das MK, Datta R, et al. Clinical profile and therapeutic response of scrub typhus in children: A recent trend from Eastern India. J Trop Pediatr [Internet]. 2019;65:139–46. Available from: <https://academic.oup.com/tropej/article/65/2/139/5001773>
11. Behera SP, Kumar N, Singh R, Deval H, Zaman K, Misra B, et al. Molecular Detection and Genetic Characterization of Orientia tsutsugamushi from Hospitalized Acute Encephalitis Syndrome Cases during Two Consecutive Seasonals in Eastern Uttar Pradesh, India. Vector-Borne Zoonotic Dis. 2021;21:747–52.
12. Bhengsri S, Baggett HC, Edouard S, Dowell SF, Dasch GA, Fisk TL, et al. Sennetsu neorickettsiosis, spotted fever group, and typhus group rickettsioses in three provinces in Thailand. Am J Trop Med Hyg. 2016;95:43–9.
13. Bithu R, Kanodia V, Maheshwari RK. Possibility of scrub typhus in fever of unknown origin (FUO) cases: an experience from Rajasthan. Indian J Med Microbiol. 2014;32:387–90.
14. Blacksell SD, Sharma NP, Phumratanaprapin W, Jenjaroen K, Peacock SJ, White NJ, et al. Serological and blood culture investigations of Nepalese fever patients. Trans R Soc Trop Med Hyg. 2007;101:686–90.
15. Brown GW, Brown GW, Shirai A, Gan E, Bernthal P, Bernthal P. Antibodies to typhus in Eastern Nepal. Trans R Soc Trop Med Hyg. 1981;75:586–7.
16. Brown GW, Saunders JP, Singh S, Huxsoll DL, Shirai A. Single dose doxycycline therapy for scrub typhus. Trans R Soc Trop Med Hyg. 1978;72:412–6.
17. Cadigan FC, Andre RG, Bolton M, Gan E, Walker JS. The effect of habitat on the prevalence of human scrub typhus in Malaysia. Trans R Soc Trop Med Hyg. 1972;66:582–7.
18. Chansamouth V, Thammasack S, Phetsouvanh R, Keoluangkot V, Moore CE, Blacksell SD, et al. The Aetiologies and Impact of Fever in Pregnant Inpatients in Vientiane, Laos. PLoS Negl Trop Dis. 2016;10:e0004577.
19. Chao CC, Zhang Z, Belinskaya T, Chen HW, Ching WM. Leptospirosis and Rickettsial Diseases Sero-Conversion Surveillance Among U.S. Military Personnel in Honduras. Mil Med [Internet]. 2022;187:802–7. Available from: <https://doi.org/10.1093/milmed/usab120>
20. Chaudhari SP, Kalorey DR, Awandkar SP, Kurkure NV, Narang R, Kashyap RS, et al. Journey towards national institute of one health in India. Indian J Med Res. 2021;153:320–6.
21. Chheng K, Carter MJ, Emary K, Chanpheaktra N, Moore CE, Stoesser N. Correction: A prospective study of the causes of febrile illness requiring hospitalization in children in Cambodia. PLoS ONE. 2015;10:e60634–e60634.
22. Chinprasatsak S, Wilairatana P, Looareesuwan S, Chappuis F, Akkhavong K, Laferl H, et al. Evaluation of a newly developed dipstick test for the rapid diagnosis of scrub typhus in febrile patients. Southeast Asian J Trop Med Public Health. 2001;32:132–6.
23. Chrispal A, Boorugu H, Gopinath KG, Prakash JAJ, Chandy S, Abraham OC, et al. Scrub typhus: An unrecognized threat in South India - Clinical profile and predictors of mortality. Trop Doct. 2010;40:129–33.
24. De Vries SG, Van Eekeren LE, Van Der Linden H, Visser BJ, Grobusch MP, Wagenaar JFP, et al. Searching and Finding the Hidden Treasure: A Retrospective Analysis of Rickettsial Disease among Dutch International Travelers. Clin Infect Dis [Internet]. 2021;72:1171–8. Available from: <https://doi.org/10.1093/cid/ciaa091>
25. De W, Jing K, Huan Z, Qiong ZH, Monagin C, Min ZJ, et al. Scrub typhus, a disease with increasing threat in Guangdong, China. PLoS ONE. 2015;10:e0113968–e0113968.
26. Demma LJ, McQuiston JH, Nicholson WL, Murphy SM, Marumoto P, Sengebau-Kingzio JM, et al. Scrub typhus, Republic of Palau. Emerg Infect Dis. 2006;12:290–5.
27. Devamani CS, Schmidt W-P, Ariyoshi K, Anitha A, Kalaimani S, Prakash JAJ. Risk Factors for Scrub Typhus, Murine Typhus, and Spotted Fever Seropositivity in Urban Areas, Rural Plains, and Peri-Forest Hill Villages in South India: A Cross-Sectional Study. Am J Trop Med Hyg. 2020;103:238–48.
28. Elders PND, Swe MMM, Phyo AP, McLean ARD, Lin HN, Soe K, et al. Serological evidence indicates widespread distribution of rickettsioses in Myanmar. Int J Infect Dis. 2021;103:494–501.
29. Faruque LI, Zaman RU, Gurley ES, Massung RF, Alamgir ASM, Galloway RL, et al. Prevalence and clinical presentation of Rickettsia, Coxiella, Leptospira, Bartonella and chikungunya virus infections among hospital-based febrile patients from December 2008 to November 2009 in Bangladesh. BMC Infect Dis. 2017;17:141.
30. Fox-Lewis A, Hopkins J, Sar P, Sao S, Pheaktra N, Day NPJ, et al. Seroprevalence of dengue virus and rickettsial infections in cambodian children. Am J Trop Med Hyg. 2019;100:635–8.
31. Gautam R, Parajuli K, Tshokey T, Stenos J, Sherchand JB. Diagnostic evaluation of IgM ELISA and IgM Immunofluorescence assay for the diagnosis of Acute Scrub Typhus in central Nepal. BMC Infect Dis. 2020;20:138–138.
32. Gayani Tillekeratne L, Suchindran S, Ko ER, Petzold EA, Bodinayake CK, Nagahawatte A, et al. Previously derived host gene expression classifiers identify bacterial and viral etiologies of acute febrile respiratory illness in a south asian population. Open Forum Infect Dis. Oxford University Press US; 2020. p. ofaa194–ofaa194.
33. Graves S, Wang L, Nack Z, Jones S. Rickettsia serosurvey in Kimberley, Western Australia. Am J Trop Med Hyg. 1999;60:786–9.
34. Grigg MJ, William T, Clemens EG, Patel K, Chandna A, Wilkes CS, et al. Rickettsioses as major etiologies of unrecognized acute febrile illness, Sabah, East Malaysia. Emerg Infect Dis. 2020;26:1409–19.
35. Gu XL, Qi R, Li WQ, Jiao YJ, Yu H, Yu XJ. Misdiagnosis of scrub typhus as hemorrhagic fever with renal syndrome and potential coinfection of both diseases in patients in Shandong province, China, 2013–2014. PLoS Negl Trop Dis. 2021;15:e0009270–e0009270.
36. Gurung S, Pradhan J, Bhutia PY. Seasonal of scrub typhus in the North East Himalayan region-Sikkim: an emerging threat. Indian J Med Microbiol. 2013;31:72–4.
37. Hamaguchi S, Cuong NC, Tra DT, Doan YH, Shimizu K, Tuan NQ, et al. Clinical and epidemiological characteristics of scrub typhus and murine typhus among hospitalized patients with acute undifferentiated fever in Northern Vietnam. Am J Trop Med Hyg. 2015;92:972–8.
38. Hamzah IH, Saeed Nahaah, Al-Gharrawi SAR. Comparative study between serological tests and PCR for diagnosis of Rickettsial diseases in patients with fever of unknown origin. Meta Gene [Internet]. 2020;25:100762–100762. Available from: <https://www.sciencedirect.com/science/article/pii/S2214540020301171>
39. Harris PNA, Oltvolgyi C, Islam A, Hussain-Yusuf H, Loewenthal MR, Vincent G, et al. An seasonal of scrub typhus in military personnel despite protocols for antibiotic prophylaxis: doxycycline resistance excluded by a quantitative PCR-based susceptibility assay. Microbes Infect [Internet]. 2016;18:406–11. Available from: <http://dx.doi.org/10.1016/j.micinf.2016.03.006>
40. Hengbin G, Min C, Kaihua T, Jiaqi T. The foci of scrub typhus and strategies of prevention in the Spring in Pingtan Island, Fujian Province. Ann N Y Acad Sci. 2006;1078:188–96.
41. Hinjoy S, Wacharapluesadee S, Iamsirithaworn S, Smithsuwan P, Padungtod P. Zoonotic and vector borne agents causing disease in adult patients hospitalized due to fever of unknown origin in Thailand. Asian Pac J Trop Dis. 2017;7:577–81.
42. Horton KC, Jiang J, Maina A, Dueger E, Zayed A, Ahmed AA, et al. Evidence of rickettsia and orientia infections among abattoir workers in Djibouti. Am J Trop Med Hyg. 2016;95:462–5.
43. Horwood PF, Duong V, Laurent D, Mey C, Sothy H, Santy K, et al. Aetiology of acute meningoencephalitis in Cambodian children, 2010-2013. Emerg Microbes Infect. 2017;6:e35.
44. Hwang JH, Jeon M, Kim CH, Lee CS. High seroprevalence of mycoplasma pneumoniae and Chlamydia pneumoniae among scrub typhus patients in South Korea. Am J Trop Med Hyg. 2019;101:859–62.
45. Jain D, Nand N, Giri K, Bhutani J. Scrub typhus infection, not a benign disease: An experience from a tertiary care center in Northern India. Med Pharm Rep. 2019;92:36–42.
46. Jain P, Prakash S, Khan DN, Garg RK, Kumar R, Bhagat A, et al. Aetiology of acute encephalitis syndrome in Uttar Pradesh, India from 2014 to 2016. J Vector Borne Dis. 2017;54:311–6.
47. Jain P, Prakash S, Tripathi PK, Chauhan A, Gupta S, Sharma U, et al. Emergence of Orientia tsutsugamushi as an important cause of Acute Encephalitis Syndrome in India. PLoS Negl Trop Dis. 2018;12:e0006346–e0006346.
48. Jakharia A, Borkakoty B, Biswas D, Yadav K, Mahanta J. Seroprevalence of Scrub Typhus Infection in Arunachal Pradesh, India. Vector-Borne Zoonotic Dis. 2016;16:659–63.
49. Jiang J, Marienau KJ, May LA, Beecham HJ, Wilkinson R, Ching WM, et al. Laboratory diagnosis of two scrub typhus seasonals at Camp Fuji, Japan in 2000 and 2001 by enzyme-linked immunosorbent assay, rapid flow assay, and Western blot assay using outer membrane 56-kD recombinant proteins. Am J Trop Med Hyg. 2003;69:60–6.
50. Kamarasu K, Malathi M, Rajagopal V, Subramani K, Jagadeeshramasamy D, Mathai E. Serological evidence for wide distribution of spotted fevers & typhus fever in Tamil Nadu. Indian J Med Res. 2007;126:128–30.
51. Kamble S, Mane A, Sane S, Sonavale S, Vidhate P, Singh M, et al. Seroprevalence & seroincidence of Orientia tsutsugamushi infection in Gorakhpur, Uttar Pradesh, India: A community-based serosurvey during lean (April-May) & epidemic (October-November) periods for acute encephalitis syndrome. Indian J Med Res. 2020;151:350–350.
52. Katoh S, Cuong NC, Hamaguchi S, Thuy PT, Cuong DD, Anh LK, et al. Challenges in diagnosing scrub typhus among hospitalized patients with undifferentiated fever at a national tertiary hospital in northern Vietnam. PLoS Negl Trop Dis. 2019;13:e0007928–e0007928.
53. Khan SA, Murhekar MV, Bora T, Kumar S, Saikia J, Kamaraj P, et al. Seroprevalence of Rickettsial Infections in Northeast India: A Population-Based Cross-Sectional Survey. Asia Pac J Public Health. 2021;33:516–22.
54. Khan SA, Bora T, Chattopadhyay S, Jiang J, Richards AL, Dutta P. Seroepidemiology of rickettsial infections in Northeast India. Trans R Soc Trop Med Hyg. 2016;110:487–94.
55. Kim DM, Kyung JW, Chi YP, Ki DY, Hyong SK, Tae YY, et al. Distribution of eschars on the body of scrub typhus patients: A prospective study. Am J Trop Med Hyg. 2007;76:806–9.
56. Kocher C, Jiang J, Morrison AC, Castillo R, Leguia M, Loyola S, et al. Serologic evidence of scrub typhus in the peruvian Amazon. Emerg Infect Dis. 2017;23:1389–91.
57. Kumar V, Kumar V, Yadav AK, Iyengar S, Bhalla A, Sharma N, et al. Scrub Typhus Is an Under-recognized Cause of Acute Febrile Illness with Acute Kidney Injury in India. PLoS Negl Trop Dis. 2014;8:6–6.
58. Lai CH, Chang LL, Lin JN, Tsai KH, Hung YC, Kuo LL, et al. Human spotted fever group rickettsioses are underappreciated in Southern Taiwan, particularly for the species closely-related to Rickettsia felis. PLoS ONE. 2014;9:e95810–e95810.
59. Lakshmi RMMVN, Dharma TV, Sudhaharan S, Surya SMV, Emmadi R, Yadati SR, et al. Prevalence of scrub typhus in a tertiary care centre in Telangana, South India. Iran J Microbiol [Internet]. 2020;12:204–8. Available from: /pmc/articles/PMC7340608/
60. Liu YX, Feng D, Suo JJ, Xing YB, Liu G, Liu LH, et al. Clinical characteristics of the autumn-winter type scrub typhus cases in south of Shandong province, northern China. BMC Infect Dis. 2009;9:1–11.
61. Liu YX, Jia N, Xing YB, Suo JJ, Du MM, Jia N, et al. Consistency of the Key Genotypes of Orientia tsutsugamushi in Scrub Typhus Patients, Rodents, and Chiggers from a New Endemic Focus of Northern China. Cell Biochem Biophys. 2013;67:1461–6.
62. Liyanapathirana VC, Thevanesam V. Seroepidemiololgy of rickettsioses in Sri Lanka: A patient-based study. BMC Infect Dis. 2011;11:1–10.
63. Mahajan V, Guglani V, Singla N, Chander J. Spectrum of multiorgan dysfunction in scrub typhus infection. J Trop Pediatr [Internet]. 2021;67. Available from: <https://academic.oup.com/tropej/article/doi/10.1093/tropej/fmab074/6363912>
64. Maina AN, Farris CM, Odhiambo A, Jiang J, Laktabai J, Armstrong J, et al. Q fever, scrub typhus, and rickettsial diseases in children, Kenya, 2011–2012. Emerg Infect Dis. 2016;22:883–6.
65. Manjunathachar HV, Barde P, Raut C, Tiwari P, Chouksey V, Gowda K, et al. Determination of cut-off of diagnostic ELISA for Scrub typhus in endemic setup: Central India. J Vector Borne Dis [Internet]. 2021;58:90–3. <http://dx.doi.org/10.4103/0972-9062.316272>
66. Mansoor T, Fomda BA, Koul AN, Bhat MA, Abdullah N, Bhattacharya S, et al. Rickettsial infections among the undifferentiated febrile patients attending a tertiary care teaching hospital of northern India: A longitudinal study. Infect Chemother. 2021;53:96–106.
67. Mathai E, Lloyd G, Cherian T, Abraham OC, Cherian AM. Serological evidence for the continued presence of human rickettsioses in southern India. Ann Trop Med Parasitol. 2001;95:395–8.
68. Mathai E, Rolain JM, Verghese GM, Abraham OC, Mathai D, Mathai M, et al. Seasonal of scrub typhus in southern India during the cooler months. Ann N Y Acad Sci. 2003;990:359–64.
69. Maude RR, Ghose A, Samad R, de Jong HK, Fukushima M, Wijedoru L, et al. A prospective study of the importance of enteric fever as a cause of non-malarial febrile illness in patients admitted to Chittagong Medical College Hospital, Bangladesh. BMC Infect Dis. 2016;16:567–567.
70. Mayxay M, Sengvilaipaseuth O, Chanthongthip A, Dubot-Pérès A, Rolain JM, Parola P, et al. Causes of fever in rural southern Laos. Am J Trop Med Hyg. 2015;93:517–20.
71. McGready R, Ashley EA, Wuthiekanun V, Tan SO, Pimanpanarak M, Viladpai-Nguen SJ, et al. Arthropod borne disease: The leading cause of fever in pregnancy on the Thai-Burmese border. PLoS Negl Trop Dis. 2010;4:e888–e888.
72. Mina SS, Kumar V, Chhapola V. Emerging Infections in Children in North India: Scrub Typhus. J Pediatr Infect Dis. 2017;12:114–8.
73. Mittal M, Bondre V, Murhekar M, Deval H, Rose W, Verghese VP, et al. Acute Encephalitis Syndrome in Gorakhpur, Uttar Pradesh, 2016: Clinical and Laboratory Findings. Pediatr Infect Dis J. 2018;37:1101–6.
74. Mørch K, Manoharan A, Chandy S, Chacko N, Alvarez-Uria G, Patil S, et al. Acute undifferentiated fever in India: a multicentre study of aetiology and diagnostic accuracy. BMC Infect Dis. 2017;17:665.
75. Mueller TC, Siv S, Khim N, Kim S, Fleischmann E, Ariey F, et al. Acute undifferentiated febrile illness in rural Cambodia: A 3-year prospective observational study. PLoS ONE. 2014;9:e95868–e95868.
76. Murdoch DR, Woods CW, Zimmerman MD, Dull PM, Belbase RH, Keenan AJ, et al. The etiology of febrile illness in adults presenting to Patan Hospital in Kathmandu, Nepal. Am J Trop Med Hyg. 2004;70:670–5.
77. Narvencar KPS, Rodrigues S, Nevrekar RP, Dias L, Dias A, Vaz M, et al. Scrub typhus in patients reporting with acute febrile illness at a tertiary health care institution in Goa. Indian J Med Res. 2012;136:1020–4.
78. Nawab T, Srinivasa S, Reddy SP. A clinical study of rickettsial disease and its manifestations. Curr Pediatr Res. 2015;19:17–20.
79. Oberoi A, Varghese SR. Scrub typhus-an emerging entity: A study from a tertiary care hospital in North India. Indian J Public Health [Internet]. 2014 [cited 2023 Jan 25];58:281. Available from: <https://www.ijph.in/article.asp?issn=0019-557X;year=2014;volume=58;issue=4;spage=281;epage=283;aulast=Oberoi;type=0>
80. Park JH, Gill B, Acharya D, Yoo SJ, Lee K, Lee J. Seroprevalence and factors associated with scrub typhus infection among forestry workers in national park offices in South Korea. Int J Environ Res Public Health. 2021;18:1–10.
81. Paulraj PS, Renu G, Ranganathan K, Leo VJ, Veeramanoharan R. First seroprevalence report of scrub typhus from the tribal belts of the Nilgiris district, Tamil Nadu, India. Indian J Med Res [Internet]. 2021;153:503–7. Available from: <https://journals.lww.com/ijmr/Fulltext/2021/04000/First_seroprevalence_report_of_scrub_typhus_from.15.aspx>
82. Phetsouvanh R, Sonthayanon P, Pukrittayakamee S, Paris DH, Newton PN, Feil EJ, et al. The diversity and geographical structure of orientia tsutsugamushi strains from scrub typhus patients in Laos. PLoS Negl Trop Dis. 2015;9:e0004024–e0004024.
83. Phongmany S, Rolain JM, Phetsouvanh R, Blacksell SD, Soukkhaseum V, Rasachack B, et al. Rickettsial infections and fever, Vientiane, Laos. Emerg Infect Dis. 2006;12:256–62.
84. Pokhrel A, Rayamajhee B, Khadka S, Thapa S, Kapali S, Pun SB, et al. Seroprevalence and clinical features of scrub typhus among febrile patients attending a referral hospital in Kathmandu, Nepal. Trop Med Infect Dis. 2021;6.
85. Prabhakaran A, Lal S, Biswas S, Vinoth S, Asraf AS, Mittal V. Serological study of Rickettsial diseases in human and rodent population in Chittoor dist. (A.P.). J Commun Dis. 2010;42:209–13.
86. Pradeepan JA, Ketheesan N, Murugananthan K. Emerging scrub typhus infection in the northern region of Sri Lanka. BMC Res Notes. 2014;7:1–4.
87. Pradutkanchana J, Pradutkanchana S, Kemapanmanus M, Wuthipum N, Silpapojakul K. The etiology of acute pyrexia of unknown origin in children after a flood. Southeast Asian J Trop Med Public Health. 2003;34:175–8.
88. Premaratna R, Loftis AD, Chandrasena TGAN, Dasch GA, de Silva HJ. Rickettsial infections and their clinical presentations in the Western Province of Sri Lanka: a hospital-based study. Int J Infect Dis. 2008;12:198–202.
89. Raina S, Raina RK, Agarwala N, Raina SK, Sharma R. Coinfections as an aetiology of acute undifferentiated febrile illness among adult patients in the sub-Himalayan region of north India. J Vector Borne Dis [Internet]. 2018;55:130–6. Available from: <http://www.jvbd.org>
90. Ramyasree A, Kalawat U, Rani ND, Chaudhury A. Seroprevalence of Scrub typhus at a tertiary care hospital in Andhra Pradesh. Indian J Med Microbiol. 2015;33:68–72.
91. Rao PN, Van Eijk AM, Choubey S, Ali SZ, Dash A, Barla P, et al. Dengue, chikungunya, and scrub typhus are important etiologies of non-malarial febrile illness in Rourkela, Odisha, India. BMC Infect Dis. 2019;19:572–572.
92. Rathi NB, Rathi AN, Goodman MH, Aghai ZH. Rickettsial diseases in Central India: Proposed clinical scoring system for early detection of Spotted Fever. Indian Pediatr. 2011;48:867–72.
93. Rauf A, Singhi S, Nallasamy K, Walia M, Ray P. Non-respiratory and non-diarrheal causes of acute febrile illnesses in children requiring hospitalization in a tertiary Care Hospital in North India: A prospective study. Am J Trop Med Hyg. 2018;99:783–8.
94. Raychaudhuri D, Sarkar M, Roy A, Roy D, Datta K, Sengupta T, et al. COVID-19 and Co-infection in children: The Indian perspectives. J Trop Pediatr [Internet]. 2021;67. Available from: <https://academic.oup.com/tropej/article/doi/10.1093/tropej/fmab073/6363804>
95. AL, Ratiwayanto S, Rahardjo E, Kelly DJ, Dasch GA, Fryauff DJ, et al. Serologic evidence of infection with ehrlichiae and spotted fever group rickettsiae among residents of Gag Island, Indonesia. Am J Trop Med Hyg. 2003;68:480–4.
96. Richards AL, Soeatmadji DW, Widodo MA, Sardjono TW, Yanuwiadi B, Hernowati TE, et al. Seroepidemiologic evidence for murine and scrub typhus in Malang, Indonesia. Am J Trop Med Hyg. 1997;57:91–5.
97. Rizvi M, Sultan A, Chowdhry M, Azam M, Khan F, Shukla I, et al. Prevalence of scrub typhus in pyrexia of unknown origin and assessment of interleukin-8, tumor necrosis factor-alpha, and interferon-gamma levels in scrub typhus-positive patients. Indian J Pathol Microbiol. 2018;61:76–80.
98. Roberts T, Parker DM, Bulterys PL, Rattanavong S, Elliott I, Phommasone K, et al. A spatio-temporal analysis of scrub typhus and murine typhus in laos; implications from changing landscapes and climate. PLoS Negl Trop Dis. 2021;15:e0009685–e0009685.
99. Roopa KS, Karthika K, Sugumar M, Bammigatti C, Shamanna SB, Harish BN. Serodiagnosis of scrub typhus at a tertiary care hospital from southern India. J Clin Diagn Res. 2015;9:DC05–7.
100. Ruang-areerate T, Jeamwattanalert P, Rodkvamtook W, Richards AL, Sunyakumthorn P, Gaywee J. Genotype diversity and distribution of Orientia tsutsugamushi causing scrub typhus in Thailand. J Clin Microbiol. 2011;49:2584–9.
101. Sagin DD, Ismail G, Nasian LM, Jok JJ, Pang EKH. Rickettsial infection in five remote Orang Ulu villages in upper Rejang River, Sarawak, Malaysia. Southeast Asian J Trop Med Public Health. 2000;31:733–5.
102. Saha A, Sarkar S, Mondal T. Profile of pediatric vasculitides - Prospective hospital-based data from eastern India. Indian J Rheumatol. 2021;16:127–32.
103. Sankhyan N, Saptharishi LG, Sasidaran K, Kanga A, Singhi SC. Clinical profile of scrub typhus in children and its association with hemophagocytic lymphohistiocytosis. Indian Pediatr. 2014;51:651–3.
104. Saravanan N, Rajendiran P, Sankar S, Ramamurthy M, Sasimohan A, Vineeta V, et al. Detection of scrub typhus by real-time polymerase chain reaction and immunoglobulin M ELISA among patients with acute febrile illness. J Nat Sci Biol Med. 2020;11:66–71.
105. Shirai A, Brown GW, Gan E, Huxsoll DL, Groves MG. Rickettsia tsutsugamushi antibody in mother/cord pairs of sera. Jpn J Med Sci Biol. 1981;34:37–9.
106. Silpapojakul K, Chupuppakarn S, Yuthasompob S, Varachit B, Chaipak D, Borkerd T, et al. Scrub and murine typhus in children with obscure fever in the tropics. Pediatr Infect Dis J. 1991;10:200–3.
107. Singhi S, Rungta N, Nallasamy K, Bhalla A, Peter JV, Chaudhary D, et al. Tropical fevers in Indian intensive care units: A prospective multicenter study. Indian J Crit Care Med. 2017;21:811–8.
108. Singhsilarak T, Phongtananant S, Jenjittikul M, Watt G, Tangpakdee N, Popak N, et al. Possible acute coinfections in Thai malaria patients. Southeast Asian J Trop Med Public Health. 2006;37:1–4.
109. Sinha P, Gupta S, Dawra R, Rijhawan P. Recent seasonal of scrub typhus in North Western part of India. Indian J Med Microbiol [Internet]. 2014;32:247–50. Available from: <https://pubmed.ncbi.nlm.nih.gov/25008815/>
110. Somasunder VM, Akila K, Sijimol S, Senthamarai S, Sivasankari S, Anitha C, et al. Serological detection and epidemiological factors associated with scrub typhus among undifferentiated febrile illness patients in a tertiary care hospital. J Pure Appl Microbiol. 2021;15:1442–8.
111. Srinivasan S, Menon T. Molecular detection of Orientia tsutsugamushi from suspected scrub typhus cases. Indian J Pathol Microbiol. 2017;60:70–3.
112. Stephen S, Ambroise S, Gunasekaran D, Hanifah M, Sangeetha B, Pradeep J, et al. Serological evidence of spotted fever group rickettsiosis in and around Puducherry, south India - A three years study. J Vector Borne Dis [Internet]. 2018;55:144–50. Available from: <http://www.jvbd.org>
113. Strickman D, Tanskul P, Eamsila C, Kelly DJ. Prevalence of antibodies to rickettsiae in the human population of suburban Bangkok. Am J Trop Med Hyg. 1994;51:149–53.
114. Suputtamongkol Y, Suttinont C, Niwatayakul K, Hoontrakul S, Limpaiboon R, Chierakul W, et al. Epidemiology and clinical aspects of rickettsioses in Thailand. Ann N Y Acad Sci. 2009;1166:172–9.
115. Suttinont C, Losuwanaluk K, Niwatayakul K, Hoontrakul S, Intaranongpai W, Silpasakorn S, et al. Causes of acute, undifferentiated, febrile illness in rural Thailand: Results of a prospective observational study. Ann Trop Med Parasitol. 2006;100:363–70.
116. Syhavong B, Rasachack B, Smythe L, Rolain J-M, Roque-Afonso A-M, Jenjaroen K, et al. The infective causes of hepatitis and jaundice amongst hospitalised patients in Vientiane, Laos. Trans R Soc Trop Med Hyg. 2010;104:475–83.
117. Tanskul P, Linthicum KJ, Watcharapichat P, Phulsuksombati D, Mungviriya S, Ratanatham S, et al. A New Ecology for Scrub Typhus Associated with a Focus of Antibiotic Resistance in Rice Farmers in Thailand. J Med Entomol. 1998;35:551–5.
118. Tay ST, Ho TM, Rohani MY, Devi S. Antibodies to Orientia tsutsugamushi, Rickettsia typhi and spotted fever group rickettsiae among febrile patients in rural areas of Malaysia. Trans R Soc Trop Med Hyg. 2000;94:280–4.
119. Tay ST, Mohamed Zan HA, Lim YAL, Ngui R. Antibody Prevalence and Factors Associated with Exposure to Orientia tsutsugamushi in Different Aboriginal Subgroups in West Malaysia. PLoS Negl Trop Dis [Internet]. 2013;7:e2341–e2341. Available from: <https://journals.plos.org/plosntds/article?id=10.1371/journal.pntd.0002341>
120. Thakur CK, Chaudhry R, Gupta N, Vinayaraj EV, Singh V, Das BK, et al. Scrub typhus in patients with acute febrile illness: A 5-year study from India. Qjm [Internet]. 2020;113:404–10. Available from: <https://doi.org/10.1093/qjmed/hcz308>
121. Thangaraj JWV, Mittal M, Verghese VP, Kumar CPG, Rose W, Sabarinathan R, et al. Scrub typhus as an etiology of acute febrile illness in Gorakhpur, Uttar Pradesh, India, 2016. Am J Trop Med Hyg [Internet]. 2017;97:1313–5. Available from: <https://www.ncbi.nlm.nih.gov/pmc/articles/pmc5817754/>
122. Thap LC, Supanaranond W, Treeprasertsuk S, Kitvatanachai S, Chinprasatsak S, Phonrat B. Septic shock secondary to scrub typhus: Characteristics and complications. Southeast Asian J Trop Med Public Health. 2002;33:780–6.
123. Thapa S, Hamal P, Chaudhary NK, Sapkota LB, Singh JP. Burden of scrub typhus among patients with acute febrile illness attending tertiary care hospital in Chitwan, Nepal. BMJ Open. 2020;10:e034727–e034727.
124. Thiga JW, Mutai BK, Eyako WK, Ng’Ang’A Z, Jiang J, Richards AL, et al. High seroprevalence of antibodies against spotted fever and scrub typhus bacteria in patients with febrile illness, Kenya. Emerg Infect Dis. 2015;21:688–91.
125. Tiwari S, Nanda M. Seroprevalence of Scrub Typhus among Pyrexia of Unknown Origin Patients: A Study from Tertiary Care Hospital in Eastern Odisha, India. J Clin Diagn Res. 2020;14.
126. Trowbridge P, Divya P, Premkumar PS, Varghese GM. Prevalence and risk factors for scrub typhus in South India. Trop Med Int Health [Internet]. 2017;22:576–82. Available from: <https://onlinelibrary.wiley.com/doi/10.1111/tmi.12853>
127. Trung NV, Hoi LT, Dien VM, Huong DT, Hoa TM, Lien VN, et al. Clinical manifestations and molecular diagnosis of scrub typhus and murine typhus, Vietnam, 2015–2017. Emerg Infect Dis. 2019;25:633–41.
128. Tsai KH, Chung LH, Chien CH, Tung YJ, Wei HY, Yen TY, et al. Human granulocytic anaplasmosis in Kinmen, an offshore island of Taiwan. PLoS Negl Trop Dis. 2019;13:e0007728–e0007728.
129. Um J, Nam Y, Lim JN, Kim M, An Y, Hwang SH, et al. Seroprevalence of scrub typhus, murine typhus and spotted fever groups in North Korean refugees. Int J Infect Dis. 2021;106:23–8.
130. Usha K, Kumar E, Kalawat U, Siddhartha Kumar B, Chaudhury A, Sai Gopal DVR. Seroprevalence of scrub typhus among febrile patients: A preliminary study. Asian J Pharm Clin Res. 2014;7:19–21.
131. Usha K, Kumar E, Kalawat U, Siddhartha Kumar B, Chaudhury A, Sai Gopal DVR. Molecular detection of scrub typhus in Tirupati, Andhra pradesh, India. J Vector Borne Dis. 2015;52:171–4.
132. Vallé J, Thaojaikong T, Moore CE, Phetsouvanh R, Richards AL, Souris M, et al. Contrasting spatial distribution and risk factors for past infection with scrub typhus and murine typhus in Vientiane city, Lao PDR. PLoS Negl Trop Dis. 2010;4:1–10.
133. Varghese GM, Abraham OC, Mathai D, Thomas K, Aaron R, Kavitha ML, et al. Scrub typhus among hospitalised patients with febrile illness in South India: Magnitude and clinical predictors. J Infect [Internet]. 2006;52:56–60. Available from: <https://www.sciencedirect.com/science/article/pii/S0163445305000319>
134. Vikram K, Agarwala P, Bhargava A, Jain Y, Jagzape T, Wasnik P. Scrub typhus and leptospirosis in rural and urban settings of central India: a preliminary evaluation. Trop Doct. 2020;50:111–5.
135. Wangrangsimakul T, Althaus T, Mukaka M, Kantipong P, Wuthiekanun V, Chierakul W, et al. Causes of acute undifferentiated fever and the utility of biomarkers in Chiangrai, northern Thailand. PLoS Negl Trop Dis. 2018;12:e0006477–e0006477.
136. Weitzel T, Acosta-Jamett G, Jiang J, Martínez-Valdebenito C, Farris CM, Richards AL, et al. Human seroepidemiology of Rickettsia and Orientia species in Chile – A cross-sectional study in five regions. Ticks Tick-Borne Dis. 2020;11:101503–101503.
137. Wi YM, Woo HI, Park D, Lee KH, Kang CI, Chung DR, et al. Severe fever with thrombocytopenia syndrome in patients suspected of having scrub typhus. Emerg Infect Dis. 2016;22:1992–5.
138. Win AM, Nguyen YTH, Kim Y, Ha NY, Kang JG, Kim H, et al. Genotypic heterogeneity of Orientia tsutsugamushi in scrub typhus patients and thrombocytopenia syndrome co-infection, Myanmar. Emerg Infect Dis [Internet]. 2020;26:1878–81. Available from: <https://pubmed.ncbi.nlm.nih.gov/32687023/>
139. Yaqoob S, Siddiqui AH, Shukla P. Scrub typhus: A Neglected tropical Disease and a potential threat in North India. J Pure Appl Microbiol. 2020;14:1589–93.
140. Yen NTH, Kim C, Jeong S, Jeon K, Choi H, Ro HJ, et al. Severe fever with thrombocytopenia syndrome virus infection or mixed infection with scrub typhus in South Korea in 2000-2003. Am J Trop Med Hyg. 2019;101:1096–9.
141. Yen TY, Zhang Z, Chao CC, Ching WM, Shu PY, Tseng LF, et al. Serologic Evidence for Orientia Exposure in the Democratic Republic of Sao Tome and Principe. Vector-Borne Zoonotic Dis. 2019;19:821–7.
142. Tandale, B. V. et al. Infectious causes of acute encephalitis syndrome hospitalizations in Central India, 2018–20. Journal of Clinical Virology 153, 105194, doi:10.1016/j.jcv.2022.105194 (2022).
143. Panigrahi, A. et al. Epidemiology of scrub typhus in a tertiary care hospital of Southern Odisha: a cross sectional study. Indian Journal of Medical Microbiology 42, 92-96, doi:10.1016/j.ijmmb.2022.09.005 (2023).
144. Camprubí-Ferrer, D. et al. Doxycycline responding illnesses in returning travellers with undifferentiated non-malaria fever: a European multicentre prospective cohort study. Journal of travel medicine 30, taac094, doi:10.1093/jtm/taac094 (2023).
145. Mondal, T., Sarkar, A., Rahaman, J. & Gupta, S. D. A study of scrub typhus in a medical college hospital in West Bengal, India. Biomedicine 42, 1091-1093, doi:10.51248/.v42i5.1942 (2022).
146. Mishra, V. et al. To study the prevalence of aetiologies Acute Undfifferentiated Febrile Illnesses of the patients at a Tertiary Care Centre in Uttar Pradesh, India. Journal of Population Therapeutics and Clinical Pharmacology 30, 1719-1726, doi:10.53555/jptcp.v30i17.2844 (2023).
147. Saraswati, K. et al. Scrub typhus in Indonesia: A cross-sectional analysis of archived fever studies samples. Transactions of The Royal Society of Tropical Medicine and Hygiene, trad094, doi:10.1093/trstmh/trad094 (2024).
148. Bal, M. et al. Scrub typhus associated acute kidney injury: an emerging health problem in Odisha, India. Journal of Vector Borne Diseases 58, 359-367, doi:10.4103/0972-9062.318318 (2021).
149. Senvanpan, N. et al. Longitudinal comparison of bacterial pathogen seropositivity among wet market vendors in the Lao People's Democratic Republic. One Health 17, 100618, doi:10.1016/j.onehlt.2023 (2023).
150. Manjhi, S. S., Jain, S. K., Sharma, K. & Solanki, S. Prevalence of Scrub typhus in children presenting as Acute Encephalitis Syndrome in Eastern part of Madhya Pradesh. Int J Acad Med Pharm 5, 939-943, doi:10.47009/jamp.2023.5.3.193 (2023).
151. Gonwong, S. et al. Nationwide seroprevalence of scrub typhus, typhus, and spotted fever in young Thai men. The American Journal of Tropical Medicine and Hygiene 106, 1363, doi:10.4269/ajtmh.20-1512 (2022).
152. Devamani, C. S., Prakash, J. A. J., Alexander, N., Stenos, J. & Schmidt, W.-P. The incidence of Orientia tsutsugamushi infection in rural South India. Epidemiology & Infection 150, e132, doi:10.1017/S0950268822001170 (2022).
153. Narang, R. et al. Scrub typhus in urban areas of Wardha district in central India. Indian Journal of Medical Research 156, 435-441, doi:10.4103/ijmr.IJMR_707_19 (2022).
154. Shrestha, S. et al. Clinical profile and biochemical abnormalities in Scrub Typhus: A cross-sectional study. Annals of Medicine and Surgery 84, doi:10.1016/j.amsu.2022.104903 (2022).
155. Münch, C. C.-S. et al. Multiple Orientia clusters and Th1-skewed chemokine profile: a cross-sectional study in patients with scrub typhus from Nepal. International Journal of Infectious Diseases 128, 78-87, doi:0.1016/j.ijid.2022.12.022 (2023).
156. Damodar, T. et al. Association of scrub typhus in children with Acute Encephalitis Syndrome and Meningoencephalitis, Southern India. Emerging infectious diseases 29, 711, doi:10.3201/eid2904.221157 (2023).
157. Weitzel, T. Notes from the Field: Scrub Typhus Seasonal—Los Lagos Region, Chile, January–February 2023. Morbidity and Mortality Weekly Report (MMWR) 72, doi:10.15585/mmwr.mm7227a4 (2023).
158. Bhatia, M. et al. Serological evidence of human leptospirosis in patients with acute undifferentiated febrile illness from Uttarakhand, India: A pilot study. Journal of Lab Physicians 11, 011-016, doi:10.4103/JLP.JLP_121_18 (2020).
159. Tasak, N. et al. Prevalence of and factors associated with scrub typhus exposure among the hill tribe population living in high incidence areas in Thailand: a cross-sectional study. BMC Public Health 23, 2394, doi:10.1186/s12889-023-17313-z (2023).
160. Swain, S. K., Sahu, B. P., Panda, S. & Sarangi, R. Molecular characterization and evolutionary analysis of Orientia tsutsugamushi in eastern Indian population. Archives of Microbiology 204, 221, doi:10.1007/s00203-022-02823-y (2022).
161. Faccini-Martínez, Á. A. et al. Serologic Evidence of Orientia Infection among Rural Population, Cauca Department, Colombia. Emerging infectious diseases 29, 456, doi:10.3201/eid2902.221458 (2023).
162. Kingston, H. W. et al. Rickettsial illnesses as important causes of febrile illness in Chittagong, Bangladesh. Emerging infectious diseases 24, 638, doi:10.3201/eid2404.170190 (2018).
163. Narayanappa, D., Geetha, R. & Rajani, H. S. Diagnostic accuracy of rapid antibody detection test for scrub typhus. Indian Pediatrics 60, 546-548, doi:10.1007/s13312-023-2932-z (2023).
164. Rainey, J. J. et al. Etiology of acute febrile illnesses in Southern China: Findings from a two-year sentinel surveillance project, 2017–2019. Plos one 17, e0270586, doi:10.1371/journal.pone.0270586 (2022).
165. Nanaware, N. et al. Genotypic characterization of Orientia tsutsugamushi isolated from acute encephalitis syndrome and acute febrile illness cases in the Gorakhpur area, Uttar Pradesh, India. Frontiers in Microbiology 13, 910757, doi:10.3389/fmicb.2022.910757 (2022).
166. D'Cruz, S., Perumalla, S. K., Yuvaraj, J. & Prakash, J. A. J. Geography and prevalence of rickettsial infections in Northern Tamil Nadu, India: a cross-sectional study. Scientific Reports 12, 20798, doi:10.1038/s41598-022-21191-7 (2022).
167. Arkell, P. et al. Integrated serological surveillance of acute febrile illness in the context of a lymphatic filariasis survey in Timor-Leste: a pilot study using dried blood spots. Transactions of The Royal Society of Tropical Medicine and Hygiene 116, 531-537, doi:10.1093/trstmh/trab164 (2022).
168. Chaisiri, K. et al. Risk factors analysis for neglected human rickettsioses in rural communities in Nan province, Thailand: A community-based observational study along a landscape gradient. PLoS Neglected Tropical Diseases 16, e0010256, doi:10.1371/journal.pntd.0010256 (2022).
169. Seetha, D., Nori, S. R. C. & Nair, R. R. Molecular-based study of scrub typhus in Kerala, South India from 2014 to 2021: a laboratory-based study. Comparative Clinical Pathology 32, 347-356, doi:10.1007/s00580-023-03443-8 (2023).
170. Selvabai, A. P., Shanmugam, P. & Kumaravelu, R. Prevalence of scrub typhus among patients attending a tertiary care hospital-a prospective and retrospective study. Journal of Communicable Diseases 55, 48-52, doi:10.24321/0019.5138.202324 (2023).
171. Baidya, A., Gunasekaran, D., Dhodapkar, R., Parameswaran, N. & Kaliaperumal, V. Prevalence, clinico-laboratory features, and the functional outcome of children with scrub typhus meningoencephalitis—a cohort study. Journal of Tropical Pediatrics 68, fmac077, doi:10.1093/tropej/fmac077 (2022).
172. Sinha, P., Gupta, S., Dawra, R. & Rijhawan, P. Recent seasonal of scrub typhus in North Western part of India. Indian journal of medical microbiology 32, 247-250, doi:10.4103/0255-0857.136552 (2014).
173. Shrestha, S., Pradhan, S., Shrestha, N. & Karn, M. Scrub Typhus among Febrile Patients Admitted to the Department of Medicine in a Tertiary Care Centre: A Descriptive Cross-sectional Study. JNMA: Journal of the Nepal Medical Association 61, 576, doi:10.31729/jnma.8208 (2023).
174. Sharma, S., Gupta, M., Singla, N., Lehl, S. S. & Gaba, S. Seasonal, Regional and Demographic Trends in Patients with Acute Undifferentiated Fever in Northern India. J Indian Med Assoc 120, 33-38 (2022).
175. Murali, R., Kalpana, S., Satheeshkumar, P. & Dhandapani, P. Seroprevalence and Genotypic Characterization of Orientia tsutsugamushi in Febrile Pediatric Patients Admitted in Tertiary Care Hospital of Chennai, South India. Journal of Pure and Applied Microbiology 17, 2232-2243, doi:10.22207/JPAM.17.4.20 (2023).
176. Won, E. J. et al. Under-diagnosis of vector-borne diseases among individuals suspected of having Scrub Typhus in South Korea. PLoS One 18, e0286631, doi:10.1371/journal.pone.0286631 (2023).
177. Trung, N. V. et al. Systematic surveillance of rickettsial diseases in 27 hospitals from 26 provinces throughout Vietnam. Tropical medicine and infectious disease 7, 88, doi:10.3390/tropicalmed7060088 (2022).
178. Madhup, S. K. et al. Seroprevalence of scrub typhus in patients attending Dhulikhel Hospital, Kavre. Kathmandu University Medical Journal 19, 494-498 (2021).
179. Sultan, A. et al. Scrub Typhus: An Emerging Etiology among Undiagnosed Febrile Cases in Western Part of Uttar Pradesh, India. Journal of Pure and Applied Microbiology 16, 606-612, doi:10.22207/JPAM.16.1.60 (2022).
180. Bhowmick, I. P. et al. Diagnosis of Indigenous Non-Malarial Vector-Borne Infections from Malaria Negative Samples from Community and Rural Hospital Surveillance in Dhalai District, Tripura, North-East India. Diagnostics (Basel) 12, doi:10.3390/diagnostics12020362 (2022).
181. He, J. et al. Epidemiological and clinical characteristics of scrub typhus in Guizhou Province, China: An seasonal study of scrub typhus. PLoS Negl Trop Dis 18, e0011963, doi:10.1371/journal.pntd.0011963 (2024).

Supplementary Table 1. Search words

| **Search- Pubmed** |
| --- |
| ((Scrub typhus) OR (Japanese river fever) OR (Orientia tsutsugamushi infection) OR (Rickettsia tsutsugamushi infection) OR (Bush Typhus) OR (Chigger Borne rickettsiosis) OR (Chigger borne typhus mite typhus) OR (Mite borne typhus) OR (scrub mite borne typhus) OR (scrubtyphus) OR (tsutsugamushi disease) OR (tsutsugamushi fever) OR (shop typhus)) AND (Prevalence) |
| **Search- Scopus** |
| ( ALL ( ( scrub AND typhus ) OR ( japanese AND river AND fever ) OR ( orientia AND tsutsugamushi AND infection ) OR ( rickettsia AND tsutsugamushi AND infection ) OR ( bush AND typhus ) OR ( chigger AND borne AND rickettsiosis ) OR ( chigger AND borne AND typhus AND mite AND typhus ) OR ( mite AND borne AND typhus ) OR ( scrub AND mite AND borne AND typhus ) OR ( scrubtyphus ) OR ( tsutsugamushi AND disease ) OR ( tsutsugamushi AND fever ) OR ( shop AND typhus ) ) ) AND ( ALL ( prevalence ) ) AND ( LIMIT-TO ( DOCTYPE , "ar" ) ) |
| **Search- Embase** |
| ('scrub typhus'/exp OR 'japanese river fever' OR 'o. tsutsugamushi infection' OR 'orientia tsutsugamushi infection' OR 'r. tsutsugamushi infection' OR 'rickettsia tsutsugamushi infection' OR 'bush typhus' OR 'chigger borne rickettsiosis' OR 'chigger borne typhus' OR 'mite typhus' OR 'mite-borne typhus' OR 'rickettsiosis tsutsugamushi' OR 'scrub mite-borne typhus' OR 'scrub typhus' OR 'scrubtyphus' OR 'tsutsugamushi disease' OR 'tsutsugamushi fever' OR 'shop typhus') AND ('prevalence'/exp OR 'prevalence' OR 'prevalence study') |

Supplementary Table 2. Characteristics of the selected studies

Highlighted in Red are studies with more than one study-groups

Highlighted in Red are studies with more than one study-groups

Highlighted in Red are studies with more than one study-groups

Highlighted in Red are studies with more than one study-groups

Supplementary Table 3: Proportion positivity of scrub typhus among different age-groups

| **Age-group** | **Proportion positivity** | **No. of studies** |
| --- | --- | --- |
| Children | 4.52% | 12 |
| Children and Adolescents | 4.41% | 12 |
| Adolescents and Adults | 12.20% | 23 |
| Adults | 23.88% | 29 |
| All age groups | 52.89% | 98 |

Supplementary Table 4: Studies reporting scrub typhus with co-infections

| **Coinfections** | **No. of studies** |
| --- | --- |
| Murine typhus | 8 |
| Dengue | 26 |
| Leptospirosis | 13 |
| Spotted Fever | 13 |
| Typhus Group | 4 |
| Chikungunya | 5 |
| Japanese Encephalitis | 3 |
| Q-fever | 3 |
| Malaria | 12 |
| Kala Azar | 1 |
| Brucella | 2 |
| Typhoid | 3 |
| Influenza | 1 |
| Anaplasmosis | 1 |
| Borreliosis | 1 |
| Ehrlichiosis | 1 |
| Bartonellosis | 1 |
| Chicken pox | 4 |

Supplementary Table 5: Diagnostic tests used for detecting scrub typhus among different studies

| **Testing** | **No. of studies** |
| --- | --- |
| Weil-Felix test | 21 |
| IgM ELISA | 98 |
| IgG ELISA | 40 |
| IgM/IgG dual ELISA | 10 |
| Rapid Diagnostic Tests | 20 |
| Immunofluorescence Assay | 51 |
| Indirect Immuno-Peroxidase Assay | 5 |
| Polymerase Chain Reaction | 42 |
| Quantitative Real-time PCR | 19 |

Supplementary Table 6: Strains reported from scrub typhus positive patients

| **Strain** | **Reference** |
| --- | --- |
| Karp | 4,12,26,50,53,55,62,83,139,141,142, 148,154, 158,161,162,175 |
| Gilliam | 33,44,55,62,76,100,104,105,106,110,113,141,148,154,158,161 |
| Kato | 33,104,107,108,110,141,154,158,161,163 |
| TA763 | 107,148,161 |
| Kawasaki | 55,100,110,154 |
| Boryong | 76,104,154 |
| Ikeda | 104 |
| Sido | 104 |
| SEA1 | 108 |
| SEA2 | 108 |
| SEA3 | 108 |
| LA | 108 |
| TH1 | 108 |
| TH2 | 108 |
| LC-5A | 8 |
| U176 | 168 |
| NT0707a | 168 |
| ISS-2 | 168 |
| QNamMH | 168 |
| Jin2012 | 168 |

Supplementary Table 7. Risk of bias assessment for Cross-sectional studies. AXIS TOOL

(Green= Yes, Yellow= Don’t Know, Red= No)

(Green= Yes, Yellow= Don’t Know, Red= No)

(Green= Yes, Yellow= Don’t Know, Red= No)

(Green= Yes, Yellow= Don’t Know, Red= No)

Supplementary Table 8. Risk of bias assessment for Cohort studies. NEW-CASTLE OTTAWA SCALE

**(Green= High, Red= Low)**

Supplementary Figure 1. Funnel plot


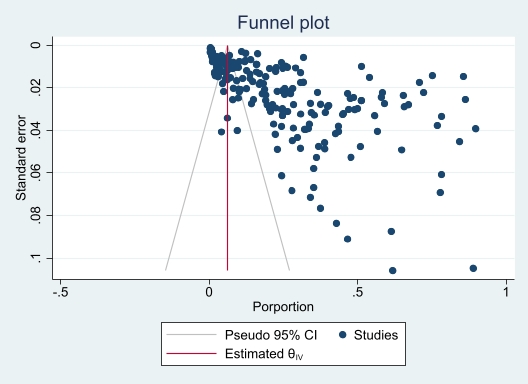


Each plotted point represents standard error and standardized mean difference of each study. The triangular region under the dotted line represents the region where 95% of the data points should lie. There is asymmetry across all the studies indicating the presence of publication bias. Most of the studies are placed in highly precise areas.

Supplementary Figure 2. Pooled prevalence of Scrub typhus among males


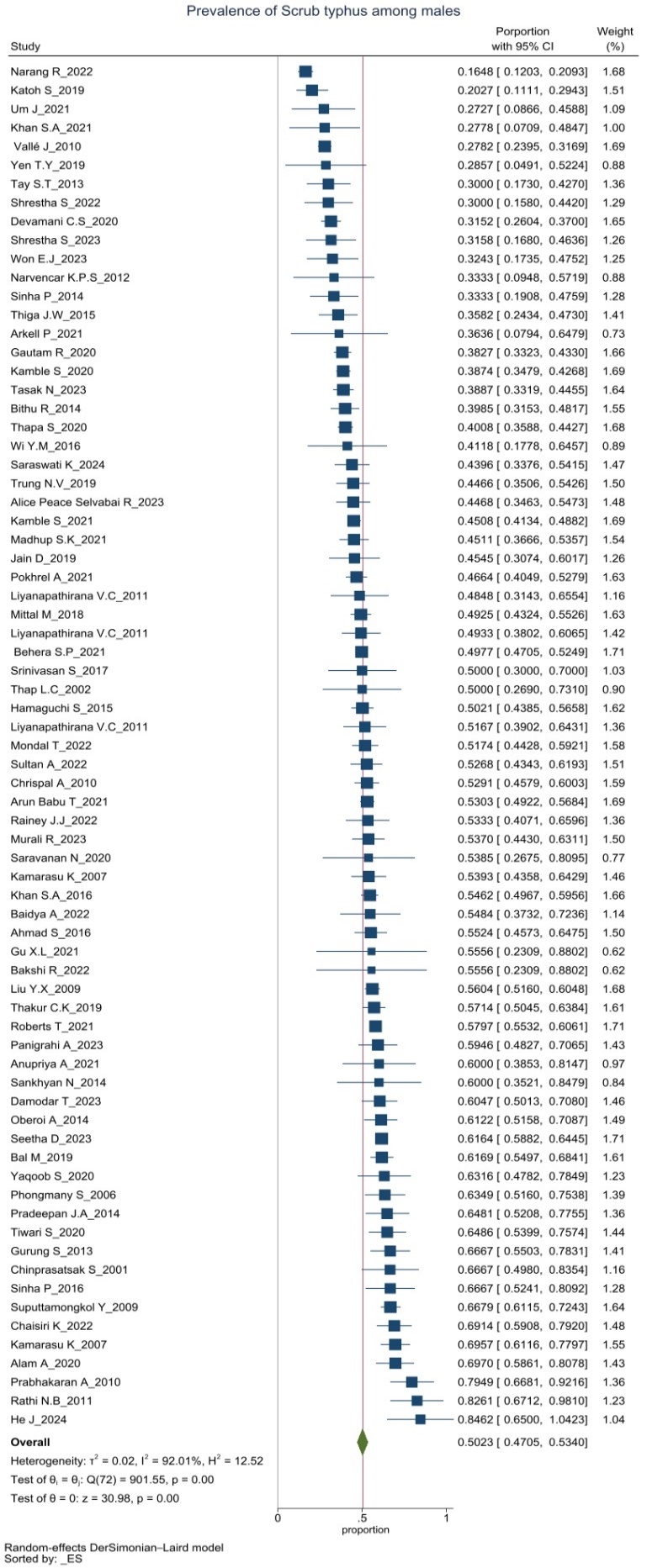


Supplementary Figure 3. Pooled prevalence of Scrub typhus among females


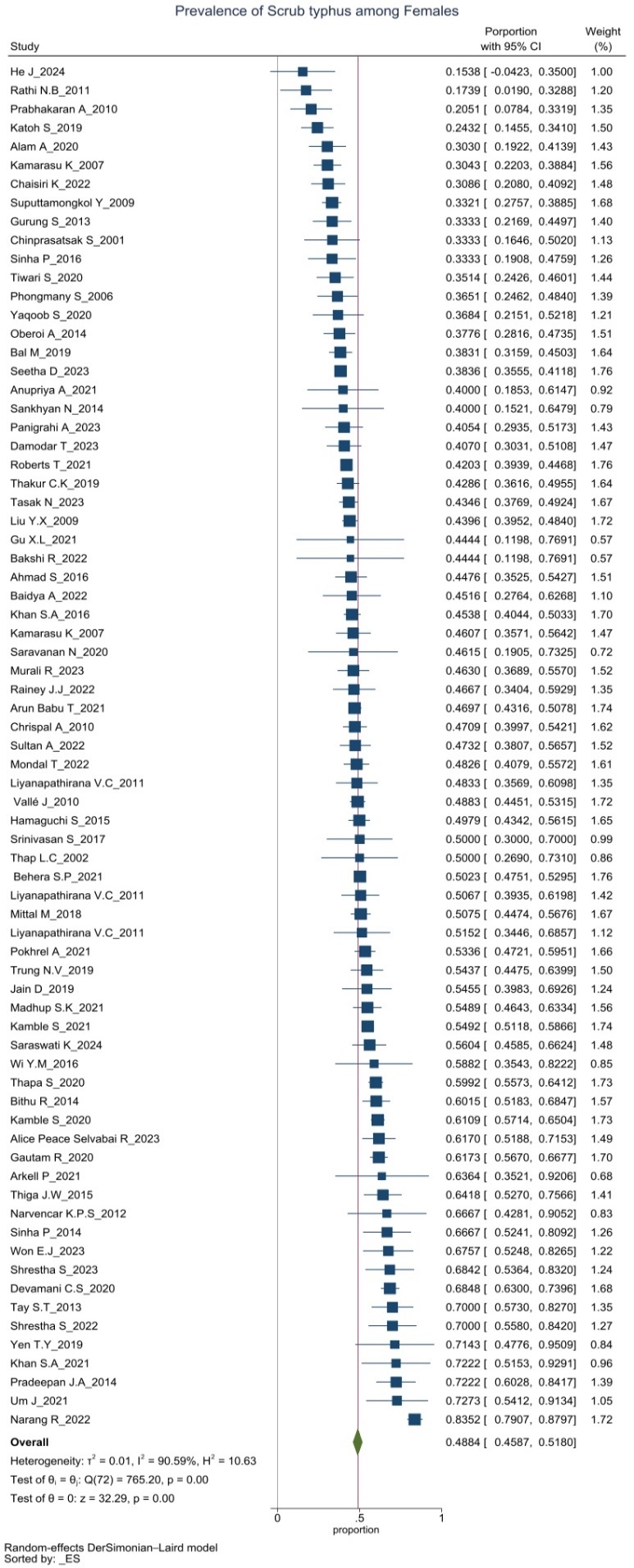


Supplementary Figure 4. Pooled prevalence of eschar among scrub typhus cases


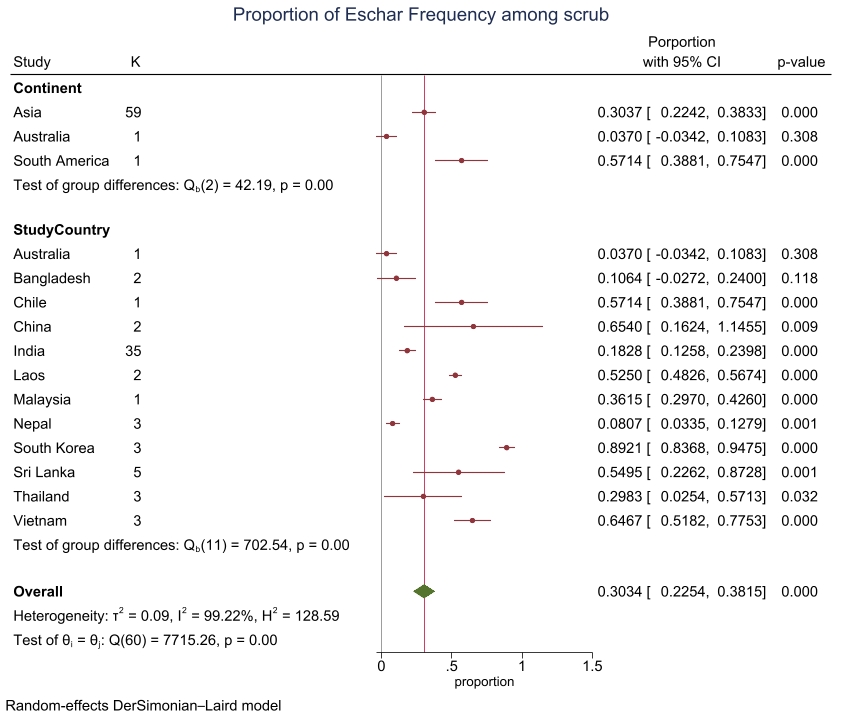


Supplementary Figure 5. Pooled prevalence of Scrub typhus during seasonal


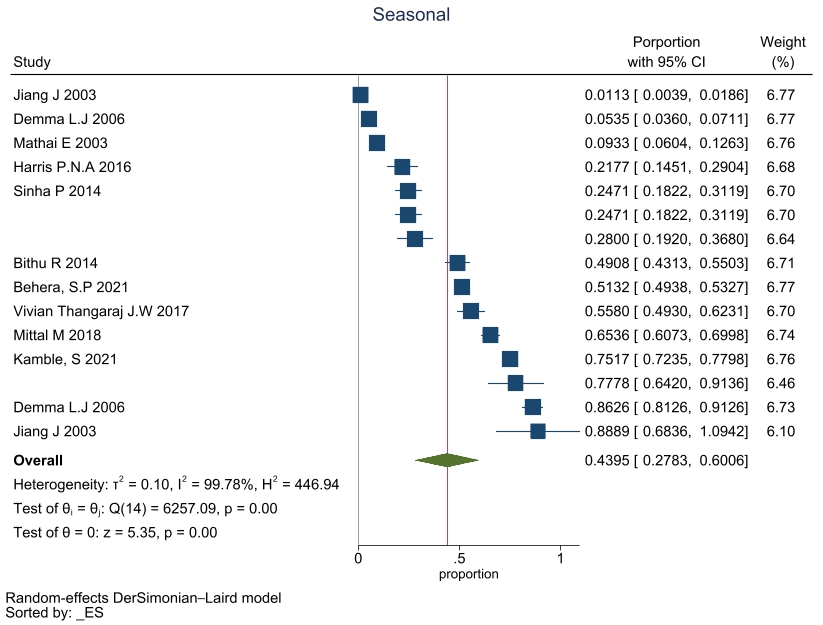


Supplementary Figure 6. Pooled prevalence of Scrub typhus during non-seasonal


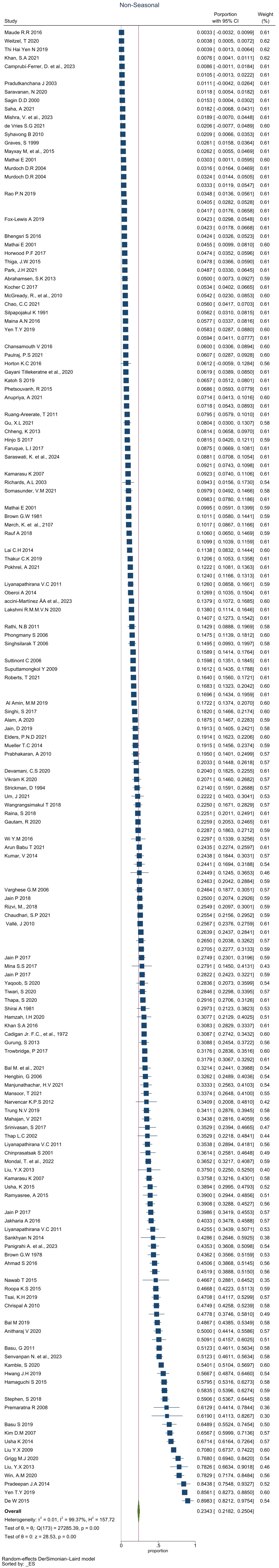


Supplementary Figure 7. Pooled prevalence of Scrub-typhus among sub-group (Population-wise)


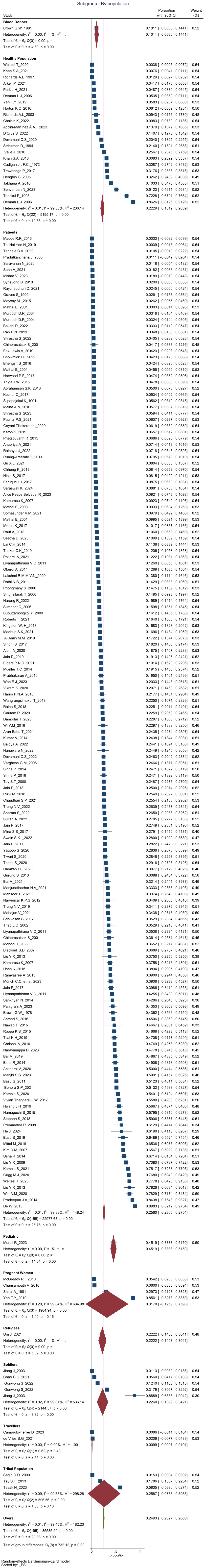


Supplementary Figure 8. Pooled prevalence of Scrub-typhus among sub-group (Study-setting: Community)


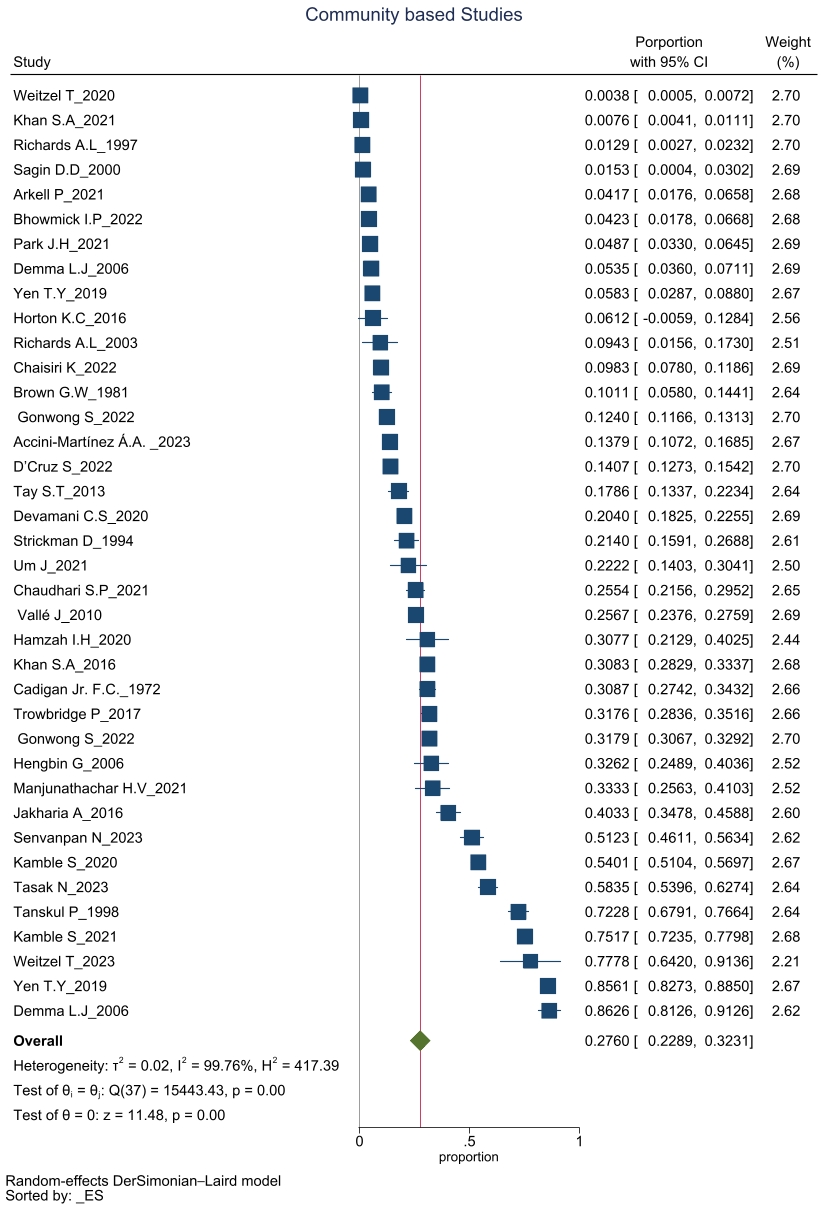


Supplementary Figure 9. Pooled prevalence of Scrub-typhus among sub-group (Study-setting: Hospital)


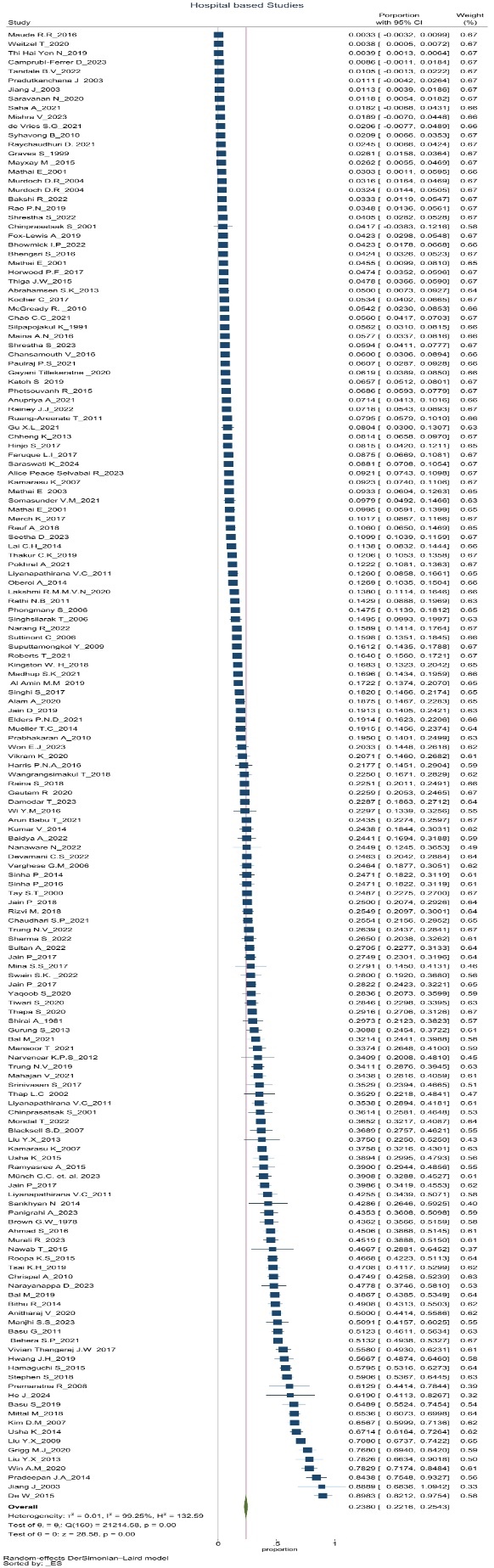


Supplementary Figure 10. Pooled prevalence of Scrub-typhus among sub-group (Study-areas)


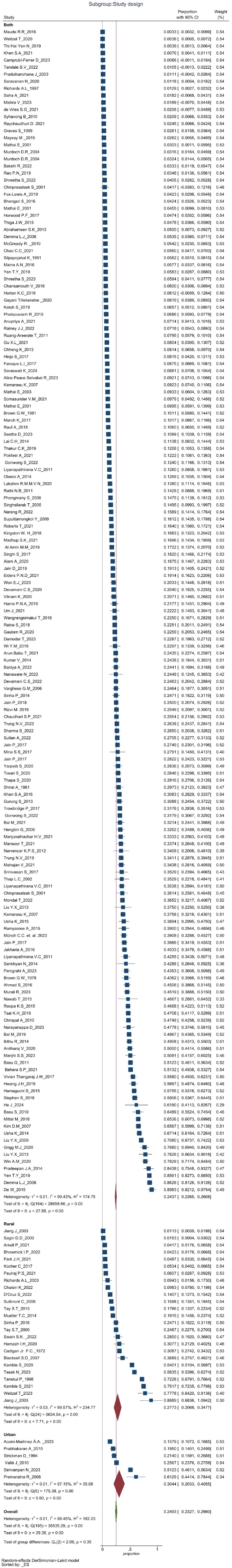


Supplementary Figure 11. Pooled prevalence of Scrub-typhus among sub-group (Country-wise)


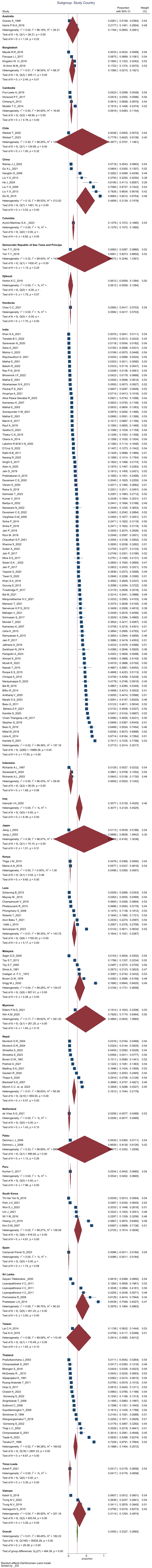


Supplementary Figure 12. Pooled seroprevalence of symptoms among Scrub typhus cases


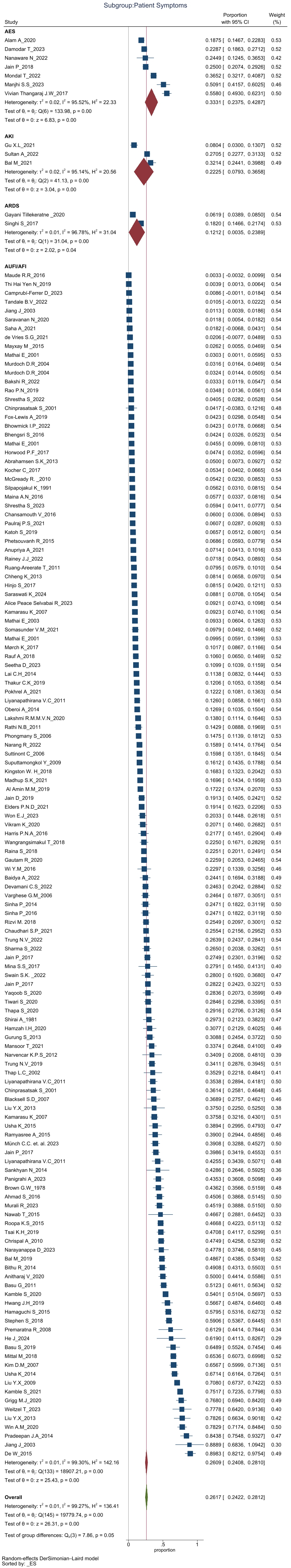


Supplementary Figure 13. Pooled prevalence of Scrub-typhus among currently infected (based on IgM)


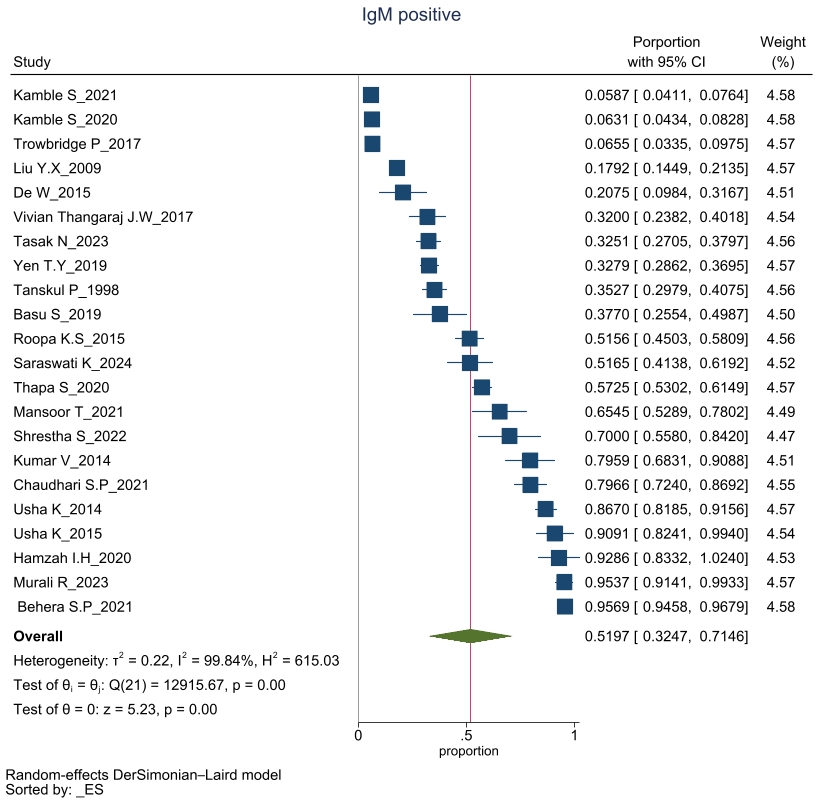


Supplementary Figure 14. Pooled prevalence of Scrub-typhus among currently infected in community-settings


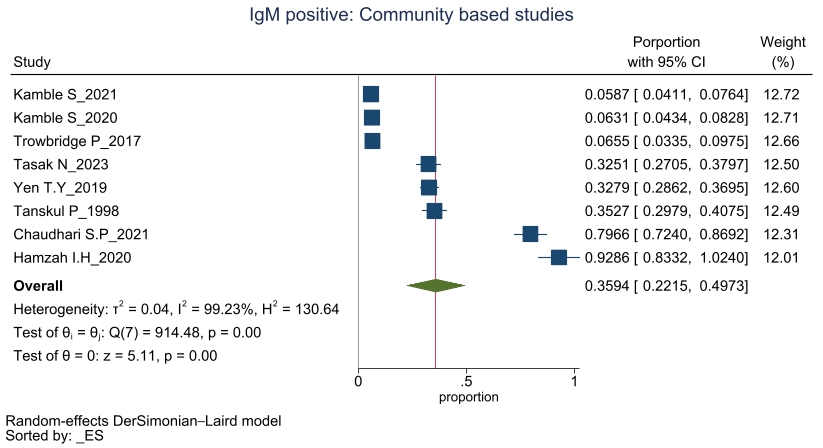


Supplementary Figure 15. Pooled prevalence of Scrub-typhus among currently infected in hospital-settings


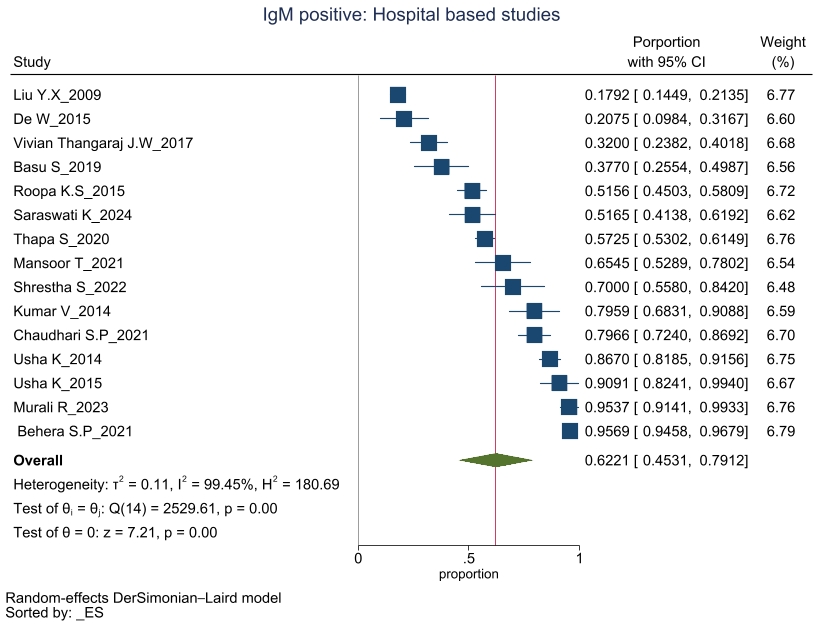


Supplementary Figure 16. Pooled prevalence of Scrub-typhus among currently infected in different populations


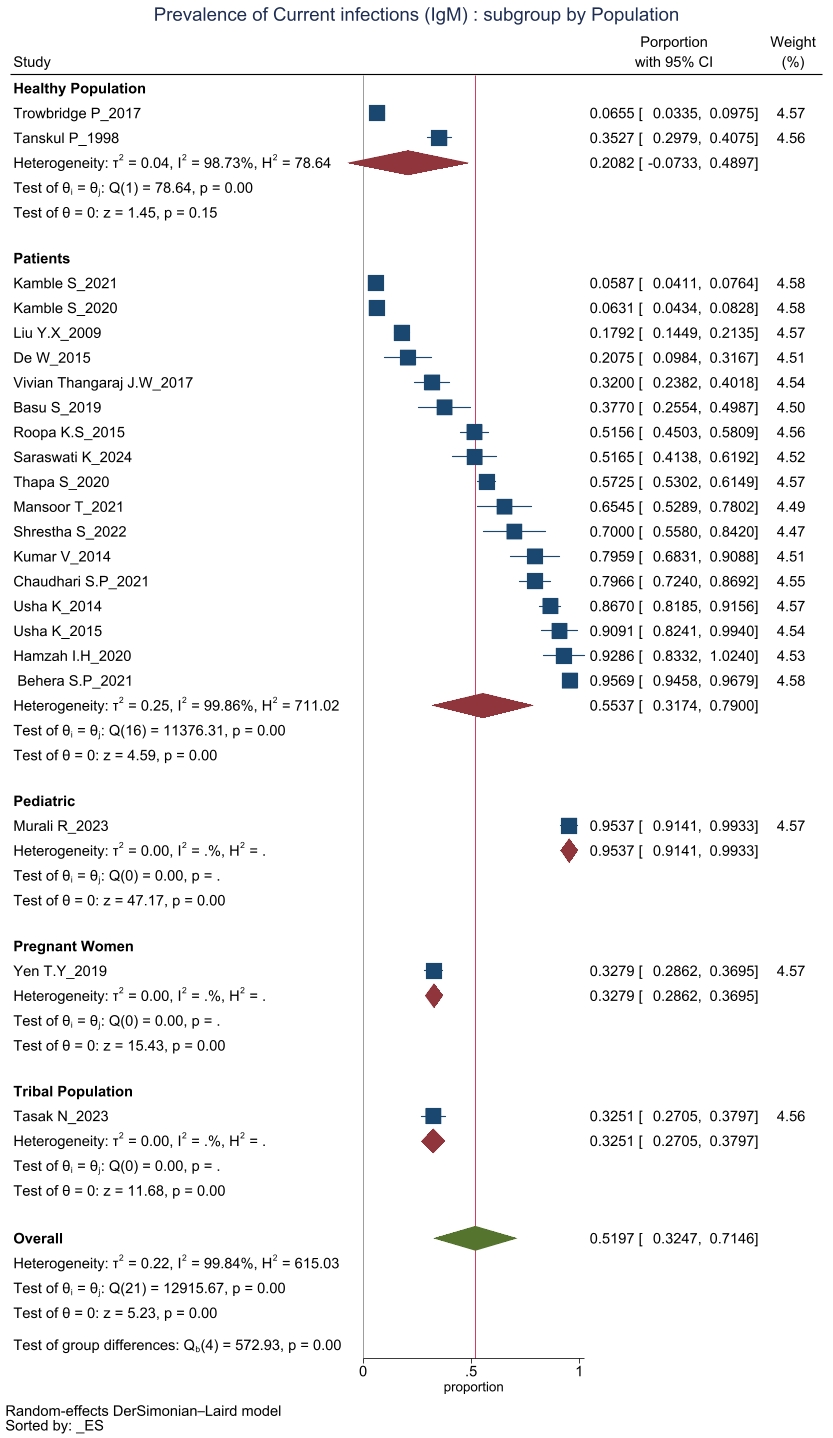


Supplementary Figure 17. Country-wise pooled prevalence of Scrub-typhus among currently infected


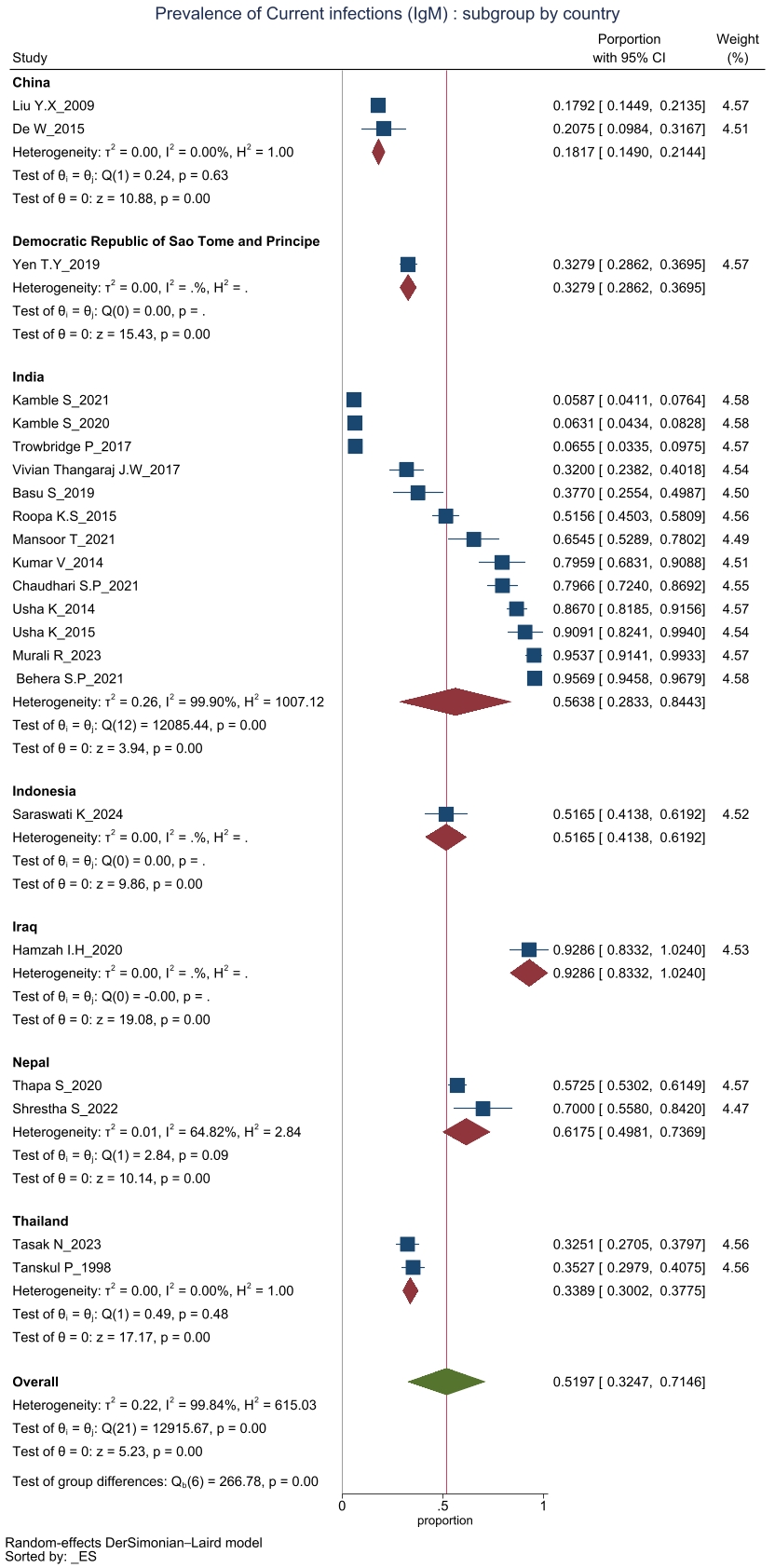


Supplementary Figure 18. Pooled prevalence of Scrub-typhus among currently infected from different study localities


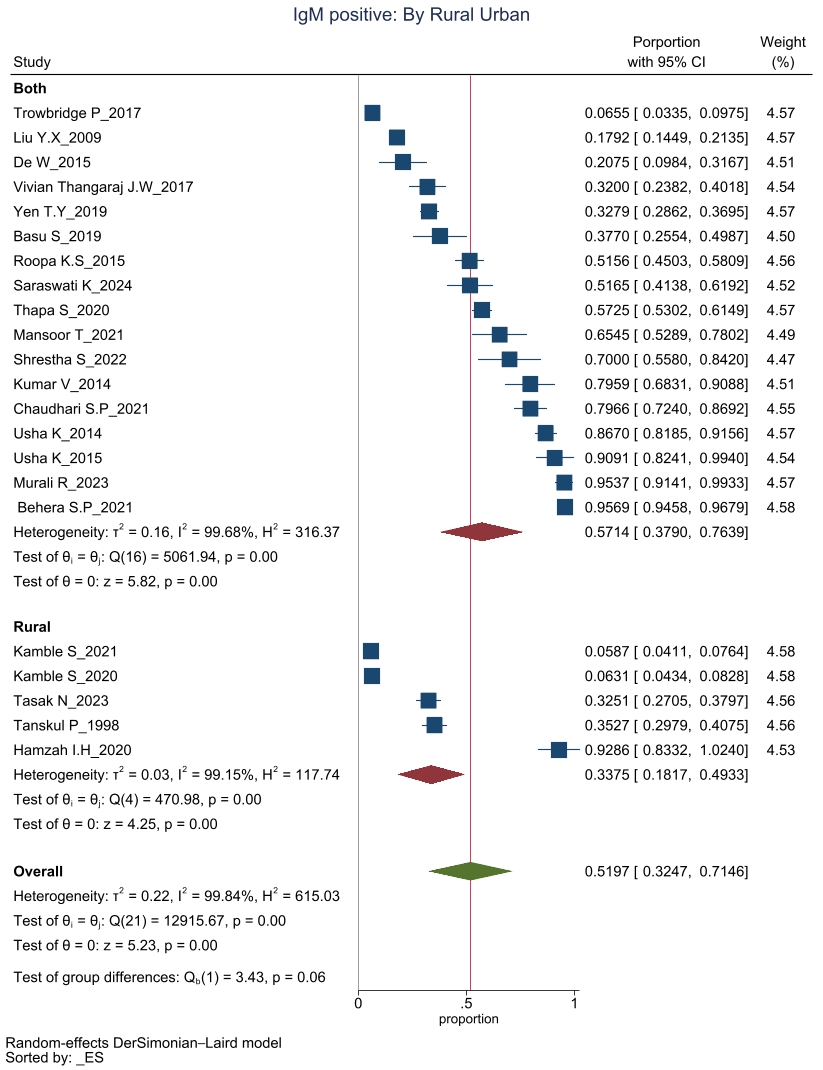


Supplementary Figure 19. Pooled prevalence of Scrub-typhus among previously exposed


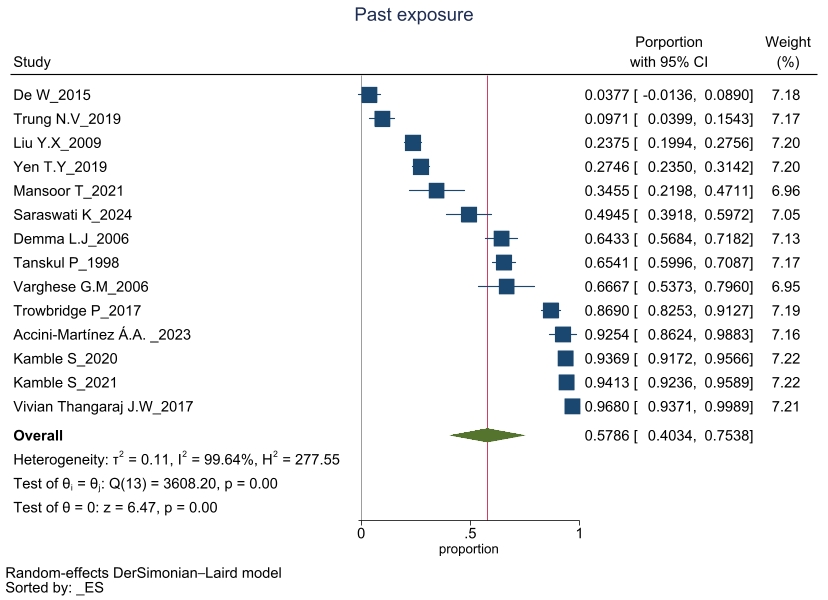


Supplementary Figure 20. Pooled prevalence of Scrub-typhus among previously exposed in community settings


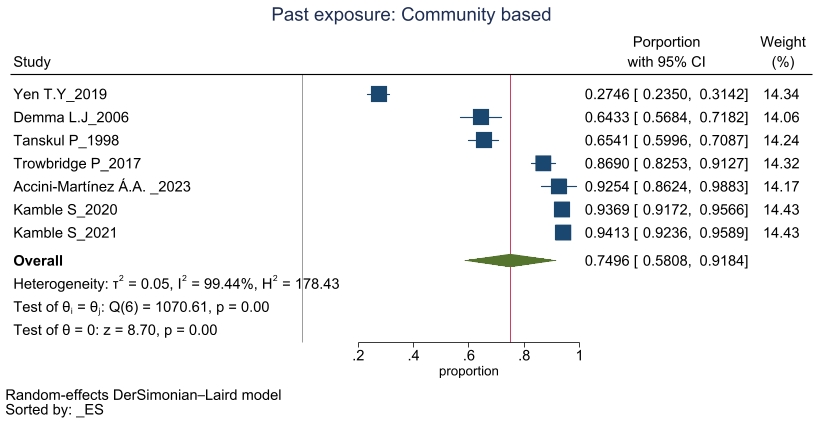


Supplementary Figure 21. Pooled prevalence of Scrub-typhus among previously exposed in hospital settings


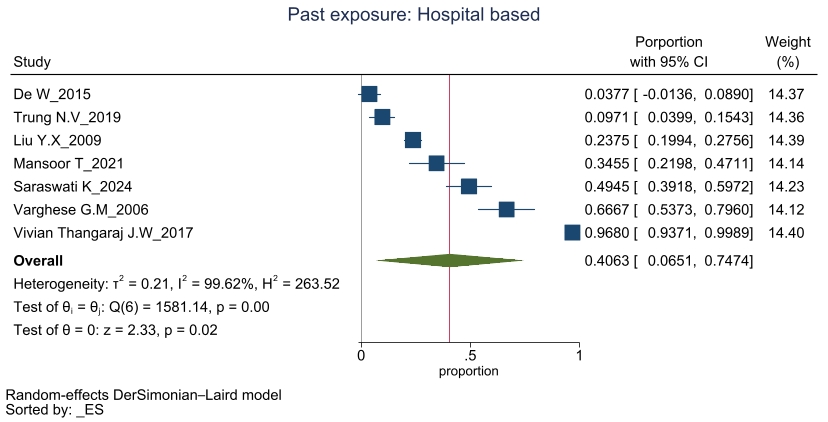


Supplementary Figure 22. Pooled prevalence of Scrub-typhus among previously exposed in different populations


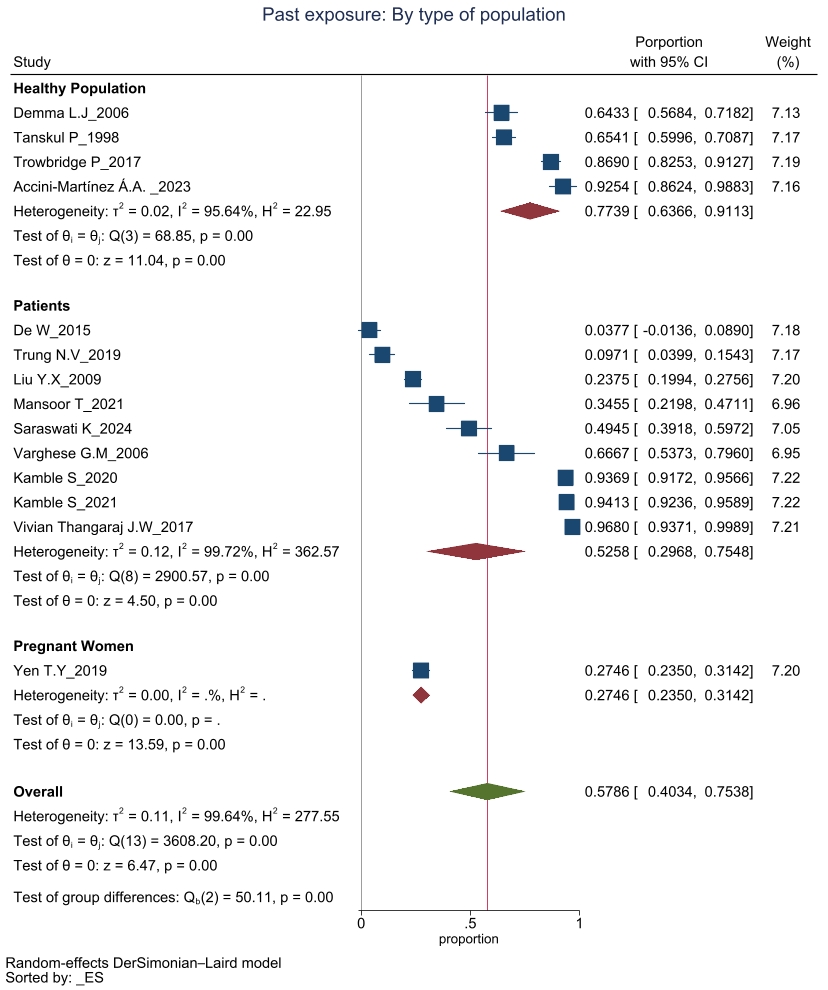


Supplementary Figure 23. Country-wise pooled prevalence of Scrub-typhus among previously exposed


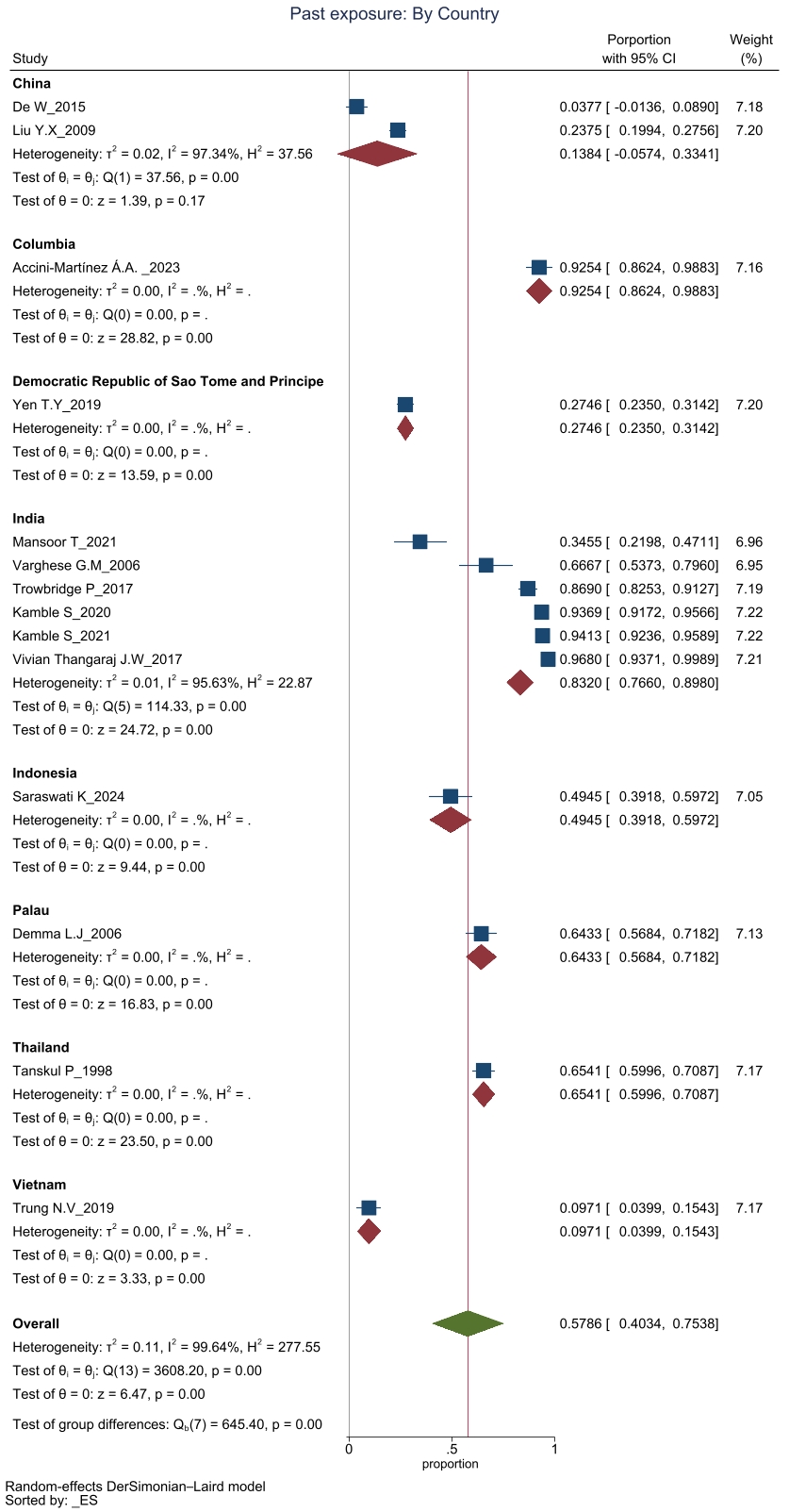


Supplementary Figure 24. Pooled prevalence of Scrub-typhus among previously exposed from different study localities


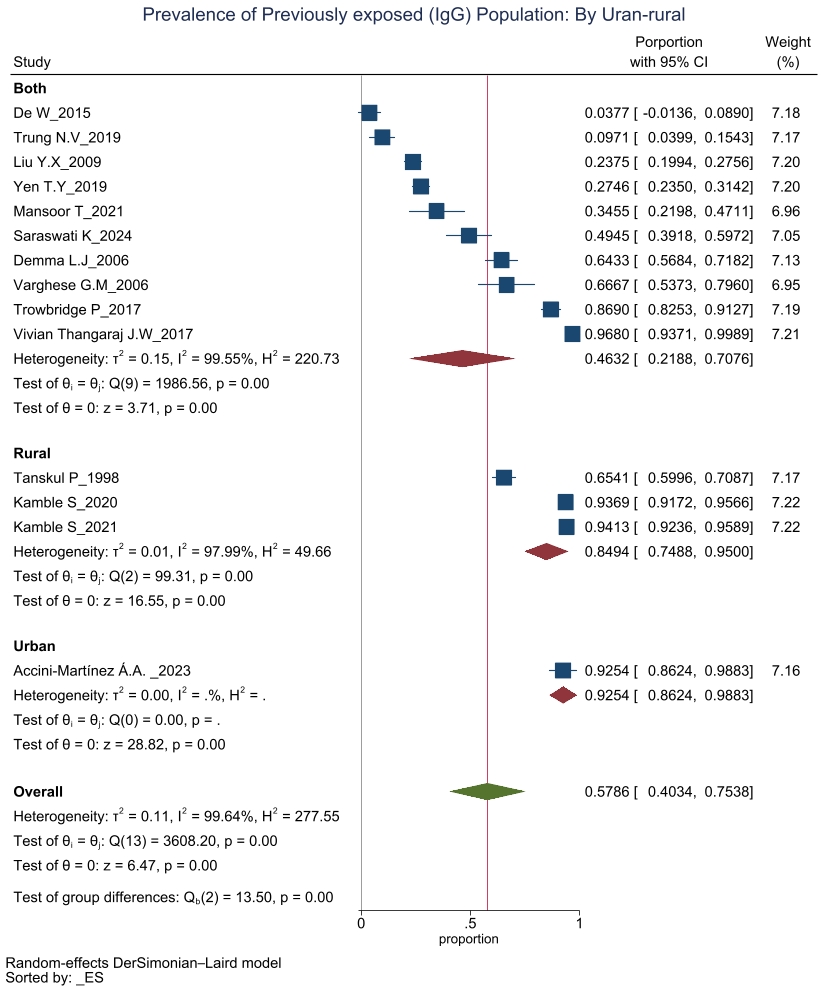


**Note on managing reviewer disagreements.**

The optimal number of reviewers for a Systematic Review and Meta-Analysis (SRMA) lacks a widely agreed-upon consensus. However, the Cochrane Handbook for Systematic Reviews of Interventions-Version 6.4, 2023 suggests involving (at least) two reviewers. Thus, our study engaged three reviewers to enhance precision and reliability.

After integrating the data of all the collected articles downloaded from the databases into Rayaan, users can activate the "BLINDOFF/ON" option. Literature screening begins with "BLIND ON," ensuring confidentiality in reviewers' decisions. This minimizes bias, as each reviewer's decision remains independent. Upon completing the screening, the "BLIND ON" option is deactivated, allowing reviewers to access decisions. Discrepancies in data are addressed collaboratively, revisiting literature for clarity. This meticulous process enhances the credibility and validity of the SRMA.
